# Clinical course of COPD patients with exercise-induced elevation of pulmonary artery pressure or less severe pulmonary hypertension presenting with respiratory symptoms and the impact of bosentan intervention—prospective, single-center, randomized, parallel-group study

**DOI:** 10.1186/s12890-024-02895-0

**Published:** 2024-02-17

**Authors:** Takeru Kashiwada, Yosuke Tanaka, Toru Tanaka, Tetsuya Okano, Yoshinobu Saito, Masahiro Seike, Mitsunori Hino, Hiroshi Kimura, Akihiko Gemma

**Affiliations:** 1 Department of Pulmonary Medicine and Oncology, Graduate School of Medicine, Nippon Medical School, 1-1-5 Sendagi, Bunkyo-Ku, Tokyo, 113-8603 Japan; 2 Department of Respiratory Medicine, Nippon Medical School, Chiba Hokusoh Hospital, 1715 Kamagari, Inzai, Chiba, 270-1694 Japan

**Keywords:** Pulmonary hypertension, COPD, Right heart catheterization, Echocardiography, Endothelin receptor antagonists

## Abstract

**Background:**

The data on bosentan were lacking for the treatment of exercise-induced elevation of pulmonary artery pressure (eePAP) or less severe PH in COPD. This study was conducted to investigate long-term efficacy and safety of bosentan for the treatment of eePAP or less severe PH in COPD.

**Methods:**

COPD patients diagnosed at this hospital as having COPD (WHO functional class II, III or IV) with eePAP or less severe PH whose respiratory symptoms were stable but remained and gradually progressed even after COPD therapy were randomly assigned in a 1:1 ratio to receive either bosentan or no PH treatment for two years and assessed at baseline and every 6 months for respiratory failure, activities of daily living (ADL), lung and heart functions by right heart catheterization (RHC), and other parameters.

**Results:**

A total of 29 patients who underwent RHC for detail examination were enrolled in the current study between August 2010 and October 2018.No death occurred in drug-treated group (*n* = 14) for 2 years; 5 patients died in untreated group (*n* = 15). Significant differences were noted between the 2 group in hospital-free survival (686.00 ± 55.87 days vs. 499.94 ± 53.27 days; hazard ratio [HR], 0.18; *P* = 0.026) and overall survival (727 days vs. 516.36 ± 55.38 days; HR, 0.095; *P* = 0.030) in all causes of death analysis, but not in overall survival in analysis of respiratory-related death. Bosentan was not associated with increased adverse events including requiring O_2_ inhalation.

**Conclusions:**

This study suggested that the prognosis for COPD patients with eePAP or less severe PH presenting with respiratory symptoms was very poor and that bosentan tended to improve their prognosis and suppress ADL deterioration without worsening respiratory failure.

**Trial registration:**

This study was registered with UMIN-CTR Clinical Trial as UMIN000004749.

First trial registration at 18/12/2010.

**Supplementary Information:**

The online version contains supplementary material available at 10.1186/s12890-024-02895-0.

## Background

Pulmonary hypertension (PH) is a common complication of chronic obstructive pulmonary disease (COPD) and is associated with worsened clinical symptoms, Exercise capacity, and prognosis [1–7].

Endothelin (ET) is one of the most potent vasoconstrictors; among its three isoforms, ET-1, ET-2 and ET-3, ET-1 is widely distributed throughout the body and is associated with pulmonary hypertension [8–10].

The presence of PH is an independent risk factor of death in patients with COPD [2, 5, 11].

Some studies report that ET is associated with elevated pulmonary arterial pressure (PAP) in COPD [12–14].

There is also a study indicating that 22% of 171 idiopathic pulmonary arterial hypertension (IPAH) patients had FEV1 /FVC < 70% [15].

A study reports that PAH-targeted therapies may be beneficial in severe PH patients with mild ventilatory impairment as with patients with WHO class I PH [16]. When mild COPD patients show severe or progressing symptoms, they may develop PAH, and PAH-targeted therapies may have potential benefits in these patients. Several endothelin receptor antagonists (ERAs) have been recommended by WHO for the treatment of class I PAH. It is indicated that ERAs may be effective in COPD patients because of their elevated ET plasma levels. However, the efficacy of specific PAH therapies such as pharmacotherapy including non-selective ERAs remains unclear, contrary to the fact that Ambrisentan, which is a selective endothelin A receptor inhibitor, should not be administered [17, 18].

Increase in PAP is well known to precede the onset of PAH symptoms [19]. In some cases, PAP is increased during exercise, although mPAP is normal at rest. Exercise-induced elevation of pulmonary artery pressure (eePAP) is considered to be an early stage of PH that could transfer to PH at rest [20, 21].

In recent years, regarding eePAP or mild PH, there have been several reports on the treatment with ERAs in (SSc)-PH [22–24].

However, the data on therapeutic intervention with ERAs in an early stage of PH are lacking and still controversial. Further studies are required to elucidate when and how therapeutic intervention should be implemented in PH associated with COPD (COPD-PH).

There is no approved drug available for COPD-PH. In advanced COPD-PH, long term oxygen therapy (LTOT) is conducted to inhibit repetitive vasospasm due to hypoxia. It remains unknown whether LTOT is beneficial in mild-to-moderate PH or exercise-induced elevation of pulmonary artery pressure in COPD patients.

Recently, hemodynamic definitions of exercise-PH were presented in 2022 ESC/ERS Guidelines for the diagnosis and treatment of pulmonary hypertension [25].

According to the current diagnostic criteria, the patient is diagnosed exercise-PH with confirmation of an mPAP/cardiac output (CO) slope > 3 mmHg/L/min between rest and exercise. It is not easy to diagnose exercise-PH.

While the aim of this prospective, randomized, parallel-group study was to investigate how the clinical course would be changed if COPD patients had exercise-induced elevation of pulmonary artery pressure (eePAP) or less severe PH and to compare the efficacy and safety of bosentan that is non-selective ERA and no treatment for 2 years in these patients.

This study also had a role as an exploratory study in order to explore the full-scale research project in the future.

## Methods

Performed as previously [26].

### Study design and methods

This was a prospective, single-center, interventional, parallel, randomized, open-label study.

### Target patient population

COPD patients (WHO functional class II, III or IV) with mild-to-moderate PH or eePAP requiring therapeutic intervention, no signs of hypoxia affecting ADL during 6-min walk test (6MWT). All patients gave written informed consent before participating in the study.

### Eligibility criteria

To be included in this study, patients had to fulfill all of the following inclusion criteria but none of the following exclusion criteria:

### Inclusion criteria

1. Patients aged 20 years or older (both sexes).
2. Patients diagnosed at this hospital as having COPD (WHO functional class II, III or IV) without hypoxia at rest or during 6MWT (to exclude those with decreased ADL and dyspnea in daily living associated with hypoxia and to minimize the influence of hypoxic pulmonary vasoconstriction [HPV] as a potential cause of PH associated with decreased partial pressure of oxygen in arterial blood [PaO2]) (PaO2 < 60 mmHg)*.
  *Including those whose hypoxia (PaO2 < 60 mmHg) had been corrected with long-term oxygen therapy (LTOT).
3. COPD patients with eePAP or less severe PH presenting with respiratory symptoms who had not required for any change of treatment within 3 months prior to study enrollment and whose symptoms were stable but remained and gradually progressed even after COPD therapy.
4. Patients with eePAP or less severe PH requiring therapeutic intervention diagnosed as assuming PAWP ≤ 15 mmHg, mPAP < 25 mmHg and mPAP on exercise (mPAPOE) ≥ 30 mmHg or 25 mmHg ≤ mPAP < 35 mmHg.
5. Inpatients and outpatients.
6. Patients who provided written informed consent before participating in this study.

### Exclusion criteria

1. Patients who had received bosentan or any other drug specific for PAH (e.g., phosphodiesterase type 5 [PDE-5] inhibitors, endothelin receptor antagonists, or prostaglandin analogs) prior to their enrollment.
2. Patients with any disease that could cause right heart overload.
  (During the entry process for this study, patients with any disease that could cause right heart overload, such as obstructive sleep apnea, cardiovascular comorbidities like HFpEF, etc., identified through procedures such as echocardiography, electrocardiogram, oxygen saturation monitoring, and imaging studies, were excluded from the study.)
3. Patients with PAWP > 15 mmHg.
4. Patients with hypoxia during 6MWT (PaO2 < 60 mmHg)*.
  * Excluded were those whose hypoxia (PaO2 < 60 mmHg) had been corrected with LTOT (i.e., those in whom LTOT is in place to ensure PaO2 > 60 mmHg both at rest and during 6MWT, who were deemed equivalent to COPD patients receiving routine therapy in clinical practice to allow them to be monitored for changes in their condition, prognosis and functional capacity for ADL).
5. Patients with a documented history of asthma, a bronchodilator response (BDR) to 400 ug salbutamol shown as a FEV1 change of ≥ 200 mL or peripheral eosinophilia > 150cells/uL, presence of typical asthma symptoms of atopy or history of IgE > 170 IU/ml.
6. Women who were pregnant or might have been pregnant, and who were lactating.
7. Patients with moderate or severe liver disorder.
8. Patients receiving treatment with cyclosporine, tacrolimus, or glibenclamide.
9. Other patients judged by the investigator to be ineligible for this study (e.g., those with any disease or condition other than COPD that might affect their ADL, such as arrhythmia, LV failure, pulmonary thromboembolism, connective tissue diseases, intervertebral disc herniation, as they were confirmed by history taking, physical examination, chest x-ray, echocardiography, lung perfusion scintigraphy, and measurements of various parameters conducted during the run-in period).

### Grouping of patients

Of all patients first diagnosed with COPD at our hospital based on the pulmonary function test and presence of COPD as confirmed by high-resolution CT findings, in order to include only COPD patients without pulmonary involvement other than COPD-related symptoms as much as possible,, and to evaluate PH caused by COPD, PAP by right heart catheterization (RHC) and right heart function by echocardiography was assessed to identify patients suggestive of progressive respiratory failure and suffering from progressive respiratory symptom.

According to the current diagnostic criteria, assuming PAWP ≤ 15 mmHg, if mPAP at rest is < 20 mmHg or without confirmation of an mPAP/cardiac output (CO) slope > 3 mmHg/L/min between rest and exercise, the patient is not diagnosed with ePH even if the mPAP on exercise (mPAPOE) is ≥ 30 mmHg [25]. In our study, however, this condition was defined as eePAP, i.e., a very mild form of PH requiring therapeutic intervention; and besides, mPAP ≤ 25 mmHg (at rest) to < 35 mmHg was defined as less severe PH needing therapeutic intervention. Since the aim of this study was to evaluate the efficacy and safety of early therapeutic intervention with bosentan in PH associated with COPD, eePAP or less severe PH requiring therapeutic intervention was diagnosed assuming PAWP ≤ 15 mmHg, if mPAP < 25 mmHg and mPAPOE ≥ 30 mmHg or 25 mmHg ≤ mPAP < 35 mmHg (severe PH was defined as mPAP ≥ 35 mmHg).

### Bosentan-treated and non-treated patients (Fig. 1)

**Fig. 1:**
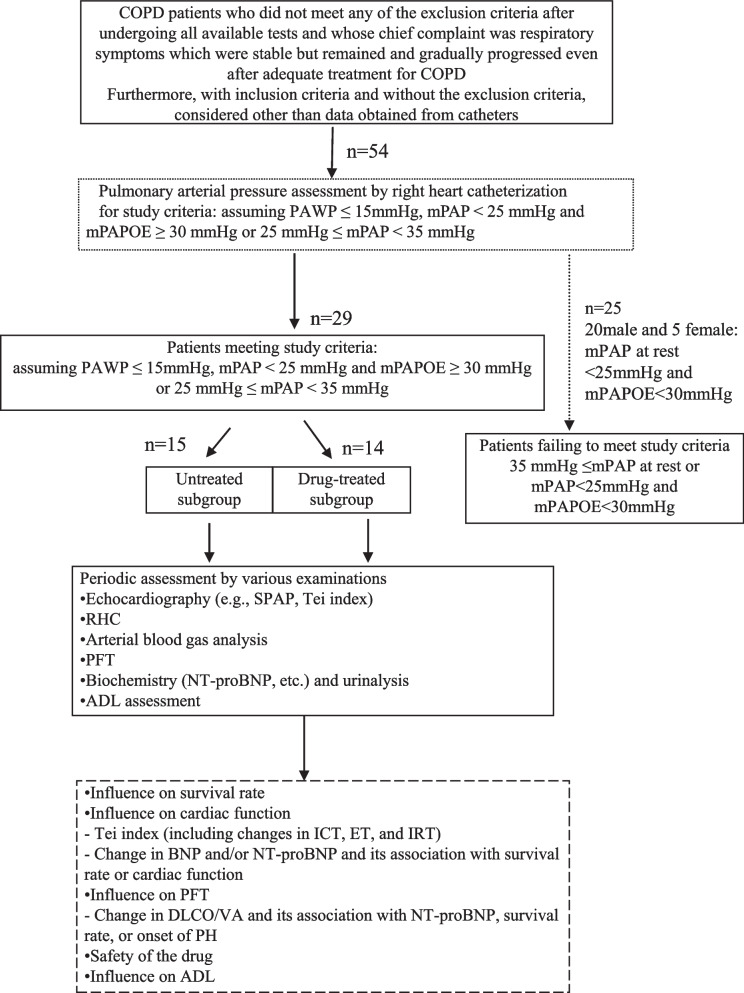
Patient flowchart. Targeted patients of patients with stable COPD who had not required any change of treatment within 3 months prior to study enrollment and with respiratory symptoms which were stable but remained and gradually progressed even after COPD therapy overtime, those with eePAP or less severe PH (no comorbidity other than COPD affecting ADL and RV strain) (mPAP < 25 mmHg and mPAPOE ≥ 30 mmHg or 25 mmHg ≤ mPAP < 35 mmHg). The study required that COPD patients be randomized to drug-treated and untreated groups to investigate their clinical course in real-world settings, with no change of treatment allowed including bosentan for 2 years or until their death as a rule, except for minimal symptomatic therapy including oxygen volume adjustments that met none of the exclusion criteria

#### Bosentan-treated and non-treated patients

All patients who met the eligibility criteria and gave informed consent prior to participating in this study were evaluated for PAP and right heart function. These patients with assuming PAWP ≤ 15 mmHg, mPAP ≥ 25 mmHg (at rest) to < 35 mmHg and/or mPAPOE ≥ 30 mmHg were stratified by mPAP. Patients were randomly allocated to either bosentan (drug-treated group) or no treatment (untreated group) by the envelope method.

Both the drug-treated and untreated group comprised those who were diagnosed at this hospital as having COPD without hypoxia (PaO_2_ > 60 mmHg) during the 6-min walk and who gave informed consent to participate in this study after PAP and right heart function assessments. This group included those with eePAP or less severe PH (mPAP ≥ 25 mmHg [at rest] to < 35 mmHg and/or mPAPOE ≥ 30 mmHg) (Fig. 1).

The study required that COPD patients be randomized to drug-treated and untreated groups to investigate their clinical course in real-world settings, with no change of treatment allowed including bosentan for 2 years or until their death as a rule, except for minimal symptomatic therapy (including oxygen volume adjustments required to ensure similar oxygen conditions among the patients), which met none of the exclusion criteria.

### Target sample size

See Supplementary data on Determination of sample size.

### Outcome measures

#### The primary outcome

##### Survival analysis

Performed as previously [26].

Hospital-free survival and overall survival were determined by the duration of survival from week 0 (start of assessment), i.e., as the date treatment started for the drug-treated group and 2 weeks after RHC for the untreated group. Even for those unable to undergo the periodic assessments due to change of their attending physician, etc., this survival analysis was continued by contacting the patient’s current physician to have his/her survival status confirmed. Patients were censored from hospital-free survival if they could no longer continue ambulatory treatment and were admitted to another hospital or if they could no longer present to our hospital for progression of respiratory failure.

#### The secondary outcomes

Echocardiography examination was carried out during the run-in period* and every 6 months thereafter**. Complete two-dimensional, pulsed-wave, color-flow echocardiography was performed using the Toshiba ultrasound system Xario (TOSHIBA MEDICAL SYSTEMS CORPORATION, Tochigi, Japan) as previously described [26–34]. (See also Supplementary document on Parameters).

Doppler measurements were carried out during the run-in period* and every 6 months**. (See also Supplementary document on Parameters with Supplementary Fig. 1).

RHC was carried out during the run-in period* and every 6 months thereafter**. (See also Supplementary document on Parameters for details on the measured hemodynamic parameters and how those parameters were measured).

### Adverse events and Other parameters

See Supplementary document on Parameters with Fig. 1, Supplementary Fig. 2 and Supplementary Fig. 3 [26–38].

### Study drug

See Supplementary document on Study drug.

### Concomitant drugs and therapies

Performed as previously [26].

Drugs allowed for use in the study included drugs intended for the treatment of the underlying disease (COPD) and drugs, other than drugs specific for PAH, for the treatment of PH as required for aggravation of PH. Drugs prohibited for use included cyclosporine, tacrolimus, glibenclamide and other drugs specific for PAH (e.g., PDE-5 inhibitors, endothelin receptor antagonists and prostaglandins) as well as any other investigational drug.

### Study period

The study was conducted for 24 months between July 2010 and December 2020 with patient enrollment lasting until December 2018 (see Supplementary document on criteria for study discontinuation).

### Statistical analysis

Data are expressed as the mean ± standard deviation (SD). Changes from baseline in individual outcome measures were compared between drug-treated and untreated patients, and analyzed for statistical significance. Analysis on paired data was performed using Mann–Whitney U test. Changes in trend over time were analyzed using the expected mean squares (EMS) method or least squares method. All statistical analyses were performed using JMP version 14sw (SAS Institute Inc., Cary, NC). A two-sided *P* value of < 0.05 was considered to indicate a statistically significant change.

## Results

### Patients

(See Supplementary Patient enrollment).

Of all, a total of 29 patients who underwent right heart catheter (RHC) for detail examination were enrolled in the current study between August 2010 and October 2018. They were all outpatients who had met the inclusion criteria. At the time of their initial presentation to our hospital, all patients were confirmed to have COPD based on pulmonary function test after inhaled bronchodilator (Fig. 1).

Of these 29 patients, 9 (including 1 female) had eePAP with mPAP < 20 mmHg at rest and 20 patients had less severe PH. All of them were randomized to receive or not to receive bosentan therapy. Of these, 14 were in the drug-treated group and the other 15 were in the untreated group; 6 in the drug-treated group and 3 in the untreated group confirmed to have eePAP diagnosed as assuming ≤ 15 mmHg, mPAPOE ≥ 30 mmHg and mPAP at rest < 20 mmHg; and 8 in the treated group and 12 in the untreated group were confirmed to have nearly eePAP based on mPAP ≤ 20 at rest to < 35 mmHg (less severe PH).

Patient demographics and characteristics were similar between the 2 groups (Table 1).

**Table 1:** Clinical characteristics of subjects with eePAP or less severe PH

	All	Untreated eePAP or less severe PH	Drug-treated eePAP or less severe PH	P*
No. (male/female)	29(26/3)	15(12/3)	14(14/0)	
Age (y.o.)	73.4±6.37	72.80 ± 6.10	74.07 ± 6.82	0.67
Height (cm)	160.51 ± 7.76	161.54 ± 9.62	159.40 ± 5.24	0.26
Weight (kg)	53.54 ± 10.10	51.75 ± 11.11	55.46 ± 8.89	0.46
No. of patients with LTOT	16	8	7	0.84
**Respiratory symptom and Activity**				
**mMRC score**	1.97 ± 1.27	2.07 ± 1.28	1.86 ± 1.29	0.77
**SGRQ score**				
Symptoms	52.48 ± 24.33	59.99 ± 25.64	44.43 ± 20.78	0.070
Activity	52.74 ± 29.03	56.87 ± 28.11	48.33 ± 30.39	0.43
Impact	30.03 ± 18.25	31.80 ± 16.73	28.13 ± 20.21	0.47
Total	42.14 ± 20.72	45.41 ± 19.80	38.64 ± 21.84	0.47
**SF36**				
Physical functioning (PF)	66.38 ± 25.60	62.00 ± 27.70	71.07 ± 23.22	0.51
Role physical (RP)	63.59 ± 28.25	57.09 ± 29.68	70.54 ± 25.88	0.26
Bodily pain (BP)	73.83 ± 27.90	69.13 ± 29.46	78.86 ± 26.26	0.41
General health (GH)	46.65 ± 13.03	47.92 ± 11.52	45.29 ± 14.80	0.86
Vitality (VT)	56.06 ± 24.85	54.19 ± 27.00	58.06 ± 23.17	0.71
Social functioning (SF)	70.26 ± 30.69	65.00 ± 33.47	75.89 ± 27.50	0.35
Role emotional (RE)	62.20 ± 31.06	52.97 ± 27.66	71.43 ± 32.47	0.11
Mental health (MH)	63.28 ± 23.23	63.67 ± 25.60	62.86± 21.37	0.69
**TMET**				
METS	3.53 ± 2.29	2.39 ±0.69	4.74 ± 2.78	0.064
**6MWT**				
6MWD	281.089 ± 127	258.57 ± 81.16	303.61 ± 161.60	0.65
**Right heart cardiography**				
mPAP (mmHg)	22.55 ± 5.24	23.87 ± 5.72	21.14 ± 4.47	0.12
mPAPOE (mmHg)	37.34 ± 7.75	39.20 ± 8.69	35.36 ± 6.32	0.20
mPAWP (mmHg)	6.72 ± 3.23	5.80 ± 2.93	7.71 ± 3.33	0.099
mRVP (mmHg)	12.97 ± 3.27	13.53 ± 3.58	12.36 ± 2.90	0.23
mRAP (mmHg)	4.48 ± 2.82	4.67 ± 3.02	4.29 ± 2.70	0.98
CO (L/min)	4.19 ± 1.24	4.36 ± 1.31	4.01 ± 1.17	0.58
CI (L/min/m^2^)	2.68 ± 0.71	2.80 ± 0.70	2.54 ± 0.73	0.34
PVR (wood)	4.55 ± 3.52	5.55 ± 4.40	3.48 ± 1.88	0.11
**Mixed venous**				
PHv	7.39 ± 0.034	7.39 ± 0.040	7.39 ± 0.029	0.59
PvCO_2_ (mmHg)	48.57 ± 6.02	49.16 ± 4.24	47.93 ± 7.61	0.22
PvO_2_ (mmHg)	36.92 ± 2.59	36.49 ± 2.78	37.37 ± 2.38	0.33
SVO_2_ (%)	69.27 ± 4.39	68.52 ± 4.46	70.06 ± 4.35	0.73
**PFT**				
FEV1(L)	1.30 ± 0.67	1.23 ± 0.68	1.39 ±0.67	0.48
FEV1%; FEV1/FVC(%)	48.61 ±20.13	48.57 ±24.21	48.65 ±15.54	0.57
%FEV1; FEV1/pred FEV1(%)	65.34 ±37.44	60.75 ±37.33	70.26 ±38.31	0.45
n (GOLD stage I/II/III/IV)	8/8/11/2	3/5/7/0	5/3/4/2	0.21
%VC; VC/pred VC (%)	91.57 ±26.14	93.67 ±26.90	89.31 ±26.11	0.74
%DLCO (%)	60.21% ± 32.18	56.84 ± 31.11	63.84 ± 34.18	0.68
**TTE**				
ET (msec)	272.5 ± 41.11	265.97 ± 32.03	279.50 ± 49.33	0.39
PAAcT (msec)	97.45 ± 21.35	94.53 ± 17.83	100.57 ± 24.89	0.35
AcT/ET	0.36 ± 0.064	0.36 ± 0.063	0.36 ± 0.067	0.82
PEP (msec)	92.19 ± 21.82	95.37 ± 22.55	88.79 ± 21.30	0.40
ICT (msec)	15.69 ± 19.60	18.60 ± 21.80	12.57 ±17.19	0.26
IRT (msec)	69.47 ± 49.99	77.90 ± 59.31	60.43 ± 37.73	0.68
TEI index	0.34 ± 0.23	0.40 ± 0.26	0.27 ± 0.17	0.21
TAPSE (cm)	2.06 ± 0.47	2.05 ± 0.48	2.08 ± 0.47	0.89
Diastolic RV area (cm^2^)	15.53 ± 4.09	15.11 ± 3.94	15.98 ±4.35	0.53
Systolic RV area (cm^2^)	9.12 ± 2.46	8.57 ± 2.52	9.71 ± 2.33	0.21
%FAC	60.81 ±14.39	63.29 ±14.99	58.14 ±13.76	0.31
Fractional Area Change (%)				
**Aortic Blood data at rest**				
pH	7.42 ± 0.037	7.42 ± 0.034	7.42 ±0.041	0.98
PCO_2_ (mmHg)	40.67 ±5.51	41.96 ±4.14	39.28 ±6.54	0.17
PO_2_ (mmHg)	75.50 ± 12.65	72.24 ± 8.04	78.99 ± 15.80	0.13
Aortic oxygen saturation (%)	94.39 ± 2.66	94.23 ± 1.44	94.56 ± 3.59	0.14
BNP (pg/ml)	34.09 ± 28.25	35.00 ± 30.38	33.11 ± 26.88	1.00
NT-proBNP (pg/ml)	138.55 ± 229.05	162.40 ± 283.52	113.00 ± 158.33	0.34
LA (mg/dl)	9.92 ± 3.61	10.55 ±4.31	9.24 ± 2.66	0.39

Data presented as mean ± SD

*P value for Wilcoxon signed-rank test to assess the difference between the untreated and drug-treated patients with ePH or less severe PH.

### Adverse events (Table 2)

**Table 2:** Adverse events observed in untreated and drug-treated patients with eePAP or less severe PH

	All	Untreated group	Drug-treated group
Exacerbation of dyspnea	20	14	6
Time to exacerbation of dyspnea **(**mean ± SD**)** (days)	200.35 ± 176.24	128.07 ± 108.96	369.00 ± 195.97
Increase of the O_2_ dose	11	8	3
Time to O_2_ dose increase **(**mean ± SE**)** (days)	168.00 ± 125.23	181.88 ± 152.84	304.00 ± 246.31
All-cause hospitalization (hospitalization-free survival days)^a^	8 (364.63 ± 238.24)	6 (339.50 ± 208.53)	2 (440.00 ± 405.88)
Hospitalization (hospital-free survival days) from respiratory-related causes	3 (230.67 ± 230.53)	2 (269.5 ± 311.83)	1 (153)
All-cause death (survival days)	7 (403.00 ± 239.83)	6 (349.00 ± 211.02	1 (727)
Death from possible respiratory-related causes (survival days)	3 (454.00 ± 325.34)	2 (317.50 ± 316.08)	1 (727)
Other adverse events	19	15^b^	4

Acute exacerbation of COPD (*n* = 2), aplastic anemia (*n* = 1), lumbar spondylolisthesis (*n* = 1)

^a^One suicide patient in the untreated group was not counted in hospitalization (hospital-free survival days) because of being treated as discontinued case

^b^Pneumoniae (*n* = 2), myocardial infarction (*n* = 2), aortic aneurysm (*n* = 2), pulmonary thrombosis-emboli (*n* = 1), sepsis due to urinary tract infection (*n* = 1), cancer (*n* = 2), cerebral infarction (*n* = 1), acute exacerbation of COPD (*n* = 1), pneumothorax with rib fracture (*n* = 1), hemosputum (*n* = 1), suicide (*n* = 1)^*^

#### Exacerbation of subjective symptoms of dyspnea(Table 2, Fig. 2a)

**Fig. 2 a:**
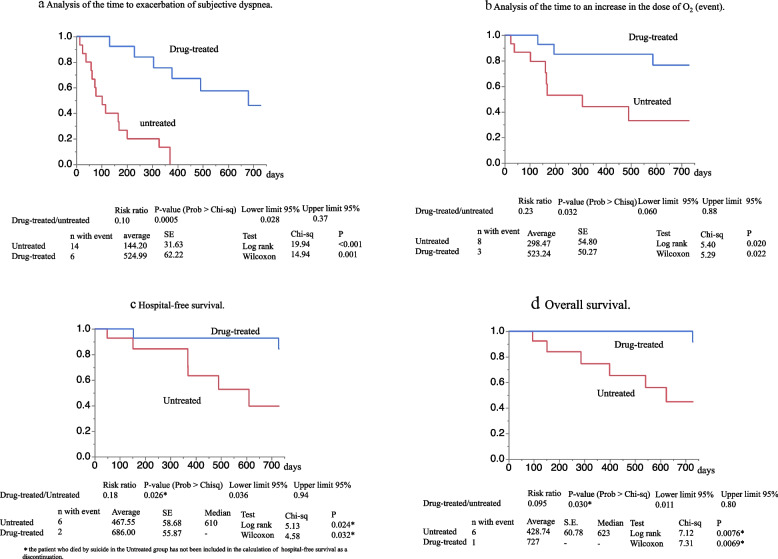
Analysis of the time to exacerbation of subjective dyspnea. Among the untreated patients with eePAP or less severe PH, the time to exacerbation of dyspnea was 128.07 ± 108.86 days (mean ± SD) in 14 of 15 patients confirmed to have experienced exacerbation of subjective symptoms of dyspnea by the obtained data. Among the drug-treated patients with eePAP or less severe PH, the time to exacerbation of dyspnea was 369.00 ± 195.97 days (mean ± SD) in 6 of 14 patients confirmed to have experienced exacerbation of dyspnea by the obtained data. The time to exacerbation of dyspnea at the time of analysis was 144.20 ± 31.63 days (mean ± SE) in the untreated group and 524.99 ± 62.22 days in the drug-treated group, and proportional hazard analysis showed that the risk ratio of the drug-treated to untreated groups was 0.10, with significant difference noted. **b** analysis of the time to an increase in the dose of O_2_ (event). Increase of the O_2_ dose: In the untreated patients with eePAP or less severe PH, the time to the dose increase was 181.88 ± 152.84 days (mean ± SD) in 8 of 15 patients confirmed to have required an increase of the dose of O_2_ by the obtained data. In the drug-treated patients with borderline or less severe PH, the time to the dose increase was 304.00 ± 246.31 days (mean ± SD) in 3 of 14 patients confirmed to have required an increase of the O_2_ dose by the obtained data. The time to O_2_ dose increase at the time of analysis was 298.47 ± 54.80 days in the untreated group and 523.24 ± 50.27 days in the drug-treated group, and the risk ratio analysis showed that the hazard ratio of the drug-treated to untreated groups was 0.23 with significant difference between the groups. The results seemed to favoring the drug-treated group. **c** Hospital-free survival. Of the 15 untreated patients with eePAP or less severe PH, 6 were confirmed to have been hospitalized (event) by the obtained (survival time analysis* in patients unable to stay at home from all causes) data with the time to hospitalization being 339.50 ± 208.53 days (mean ± SD). Of the 14 drug-treated patients with eePAP or less severe PH, 2 was confirmed to have been hospitalized by the data obtained on the cut-off date with the time to hospitalization being 440.00 ± 405.88 days. At the time of survival time analysis, hospital-free survival in the untreated group was 467.55 ± 58.68 days (mean ± SE) (median, 610 days), which was shown to be significantly different from that in the drug-treated group (6086.00 ± 55.87) by proportional hazard analysis (hazard ratio [HR] of the drug-treated to untreated groups, 0.18; *P* = 0.026). *One suicide patient in the untreated group was not counted in hospitalization (hospital-free survival days) because of being treated as discontinued case. **d** Overall survival. Of the 15 untreated patients with eePAP or less severe PH, 6 were confirmed dead (event) by the obtained (survival analysis with all-cause mortality) data with the time to event being 349.00 ± 211.02 days (mean ± SD); of the drug-treated patients with eePAP or less severe PH, 1 was confirmed dead with the time to event being 727 days. At the time of survival analysis, the time to event in the untreated group was 428.74 ± 60.78 days (mean ± SE), which was significantly different from that in the drug-treated group by proportional hazard analysis (HR of the drug-treated to untreated groups, 0.095; *P* = 0.030)

Of the 15 untreated patients, 14 were confirmed to have experienced exacerbation of subjective symptoms of dyspnea based on the obtained data, with the time to exacerbation of dyspnea being 128.07 ± 108.86 days (mean ± SD**)**. Of the 14 drug-treated patients, 6 were confirmed to have experienced exacerbation of dyspnea based on the obtained data, with the time to exacerbation being 369.00 ± 195.97 days (mean ± SD**)**. Proportional hazard analysis showed that the risk ratio of the drug-treated group to the untreated group was 0.10 with with significant difference(*p* = 0.0005).

*Details on confidence intervals for hazard ratios, etc., can be found in the figures.

##### Increase of O_2_ dose (Table 2, Fig. 2b)

In this clinical study, with the aim of minimizing the impact on pulmonary arterial pressure, a minimum oxygen dose was administered to maintain a PaO2 of 60 mmHg or above in all patients.

Of the 15 untreated patients, 8 were confirmed to have required an increase of the O_2_ dose based on the obtained data. Of the 14 drug-treated patients, 3 were confirmed to have required an increase of the O_2_ dose based on the obtained data. The risk ratio analysis showed that the hazard ratio of the drug-treated group to the untreated group was 0.23, with the time to O_2_ dose increase being 298.47 ± 54.80 days in the untreated group versus 523.24 ± 50.27 days in the drug-treated group, which was significantly different follow the fact that the results favored the drug-treated group (the drug-treated group to the untreated group risk ratio 0.23, *p* = 0.032).

*Details on confidence intervals for hazard ratios, etc., can be found in the figures.

##### Hospital-free survival (Table 2, Fig. 2c)

Of the 15 non-treated patients, 6 (respiratory failure in 2, bleeding caused by cervical cancer, myocardial infarction, aortic aneurysm and traumatic pneumothorax with rib fracture in one each) were confirmed hospitalized.

In contrast, of the 14 drug-treated patients, 2 (leg strength declines associated with breathlessness in 1, aplastic anemia in 1) were confirmed hospitalized.

When survival time analysis was conducted in patients unable to stay at home from all causes*, hospital-free survival was 467.55 ± 58.68 days (mean ± SE) (median, 610 days) in the untreated group, which was significantly different from that in the drug-treated group (686.00 ± 55.87 days) as assessed by proportional hazard analysis (hazard ratio of the drug-treated group to the untreated group, 0.18, *P* = 0.026; log-rank test, *P* = 0.024; and Wilcoxon test, *P* = 0.032).

*One suicide patient in the untreated group was not counted in hospitalization (hospital-free survival days) because of being treated as discontinued case.

*Details on confidence intervals for hazard ratios, etc., can be found in the figures.

##### Overall survival (Table 2, Fig. 2d)

Of the 15 untreated patients, 6 were confirmed dead (event) based on the obtained data. The causes of death included respiratory-related death (*n* = 2), myocardial infarction (*n* = 1), bleeding caused by cervical cancer (*n* = 1), sepsis caused by urinary tract infection (*n* = 1), and suicide (*n* = 1).

Of the 14 bosentan-treated patients with borderline or less severe PH, 1 patient was confirmed dead (sudden death possibly due to aplastic anemia or respiratory failure) with the time to event being 727 days.

At the time of survival analysis in all causes of death, the time to event was 482.75 ± 60.78 days (mean ± SE) in the untreated group, which was shown to be significantly different from that in the drug-treated group as assessed by proportional hazard analysis (hazard ratio of the drug-treated group to the untreated group, 0.095, *P* = 0.030; log-rank test, *P* = 0.0076; and Wilcoxon test, *P* = 0.0069).

However, overall survival in analysis of respiratory-related death did not show a significant difference between the two groups.

*Details on confidence intervals for hazard ratios, etc., can be found in the figures.

##### Clinical course

See Supplementary Clinical course

The clinical course in the untreated group was much poorer than in the treated group. Only quite a few patients could be assessed after month 12 in the current study excluding those patients with 1MET indicating bedridden and 6MWT = 0 m during the period since recording hospital-free survival (i.e., bedridden) and with 0MET indicating death and 6MWT = 0 m during the period since recording overall survival.

In both the treated and untreated groups, there were no instances of transaminase elevation requiring the discontinuation or inability to administer bosentan in this study.

### Lung function and RHC

#### Drug-treated patients

Compared with baseline (Table 1), significant, but slight decrease was noted in FEV1 and FEV1% at months 12, 18, and 24. However, these changes may be age-related, because there was no significant difference in %FEV for 2 years. (See Supplementary Fig. 4).

Compared with baseline (Table 1), there was a significant decrease in mPAP at months 6 and 18 (mean difference; -2.72 at month 6; *P* = 0.022, -2.56 at month 18; *P* = 0.030), and a decreasing trend at month 12 although no significant difference was noted (mean difference; -3.18; *P* = 0.063; *R* = 0.58). A similar trend was observed for PVR (-0.83 at month 6; *P* = 0.077; *R* = 0.53, -1.26 at month 12; *P* = 0.0086; *R *= 0.68; -0.94 at month 18; *P* = 0.079; *R* = 0.44). Compared with baseline, there was a significant improvement in cardiac output at month 6 and at month 12 (mean difference; + 0.74 at month 6; *P* = 0.029; *R* = 0.76, + 1.48 at month 12; *P* = 0.0033; *R* = 0.50), but no significant change was noted from baseline to month 18 and month 24. (See Supplementary Fig. 5a).

On the other hand, in the untreated group, there was no significant change at month 6 from the baseline in lung function and RHC (mean difference: FEV1: -0.20; *P* = 0.45; R =—0.060. FEV1%: -8.63; *P* = 0.21; *R* =—0.47. %FEV1: -6.96; *P* = 0.51; *R* =—0.18. %DLCO: + 3.48; *P* = 0.74; *R* =—0.32. mPAP: + 0.44; *P* = 0.85; *R* =—0.37. PVR: -0.62; *P* = 0.36; *R* =—0. 60. CO: + 0.078; *P* = 0.87; *R* =—0.35.) (See Supplementary Fig. 5b).

As mentioned previously, the clinical course in the untreated group was clearly poorer and thus it was unable to conduct a valuable analysis after month 12.

### Activity

**Fig. 3:**
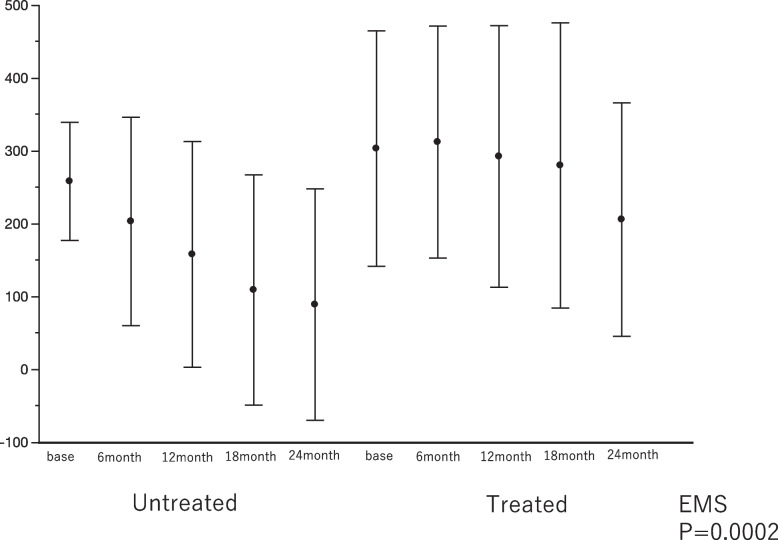
Comparison of change in 6MWD between drug-treated and untreated patients with eePAP or less severe PH**.** Repeated measures analysis of 6 min walk distance (6MWD) data using the EMS method with the standard least squares test showed a significant difference between the untreated and drug-treated patients with eePAP or less severe PH in the change from baseline to month 12 (*P* = 0.0002)

Repeated measures analysis of 6MWT data based on the EMS method using the standard least squares test showed a significant difference favoring the drug-treated group in the change in activity ability in daily living (*P* < 0.0002) (Fig. 3). The similar results were also founded in TMET data (Supplementary Table 1.)

In particular, when compared with baseline data, 6MWD showed a significant exacerbation observed in the treated group at month 24 (mean difference:-131.71 m; *P* = 0.0062). In the untreated group, there was a significant decrease in 6MWD at month 12 and thereafter (-123.50 m at month 12; *P* = 0.025, -156.50 m at month 18; *P* = 0.0073; and -177.22 m at month 24; *P* = 0.0054).

Thus, taken together, the study findings suggest that decline in physical ability and maximal exercise tolerance may have been delayed in the drug-treated group compared to the untreated group.

### Results for other assessment parameters

(See Supplementary results for other Parameters with Supplementary Fig. 6).

There was no significant change over time for other than echocardiographic assessment of right ventricular (RV) function in all patients and both groups. However, it was difficult to draw any conclusion on changes over time, because the clinical course in the untreated patients with eePAP or less severe PH was very poor and the number of these patients was small due to the increased number of those with hospital-free survival recorded.

## Discussion

COPD has long been known to be a disease with very poor prognosis [38, 39]. PH is a common complication of COPD [4, 16] and elevated PAP is shown to be associated with poor prognosis [1, 2, 5, 6, 16, 40, 41].

Several ERAs have been recommended by WHO for the treatment of class I PAH. It is indicated that ERAs may be effective in COPD patients because of their elevated ET plasma levels. However, the efficacy of specific PAH therapies such as pharmacotherapy including ERAs remains unclear [17, 18].

There is no approved drug available for COPD-PH.

According to the current diagnostic criteria, the patient is not diagnosed exercise-PH, even if the mPAPOE is ≥ 30 mmHg without confirmation of an mPAP/cardiac output (CO) slope > 3 mmHg/L/min between rest and exercise [25]. It is not easy to diagnose exercise-PH, and therapeutic intervention for PH can be delayed. The aim of this study was to investigate how the clinical course would be changed if a COPD patient had exercise-induced elevation of pulmonary artery pressure; eePAP or less severe PH and to compare the efficacy and safety of bosentan and no PAH treatment for 2 years in eePAP or mild-to-moderate PH patients with COPD presenting with respiratory symptoms which were stable but remained and gradually progressed after adequate COPD therapy. Furthermore, recognizing the difficulty of conducting an evaluation of exercise-induced pulmonary hypertension (ePH) using the standardized method in actual clinical practice [23], in this study, we assessed the increase in pulmonary arterial pressure (PAP) during right heart catheterization (RHC) by applying exercise loads simulating the daily activities of patients. Using the same method we previously reported [26], we evaluated the exercise-induced elevation of PAP in COPD patients during RHC, assuming exercise loads representative of the daily life of patients.

The current study showed that the clinical course in eePAP or mild-to-moderate PH patients with COPD was clearly poor, when the conventional COPD treatment without using bosentan was conducted.

There is a study reporting that COPD patient with PH had very poor prognosis in terms of 3-year survival rates. Considering that this study included a large number of patients with severe PH and that specific PAH therapies such as pharmacotherapy including ERAs were used in 74% of the patients, our study involving COPD patients with eePAP or less severe PH would raise a question again that these patients had very poor prognosis if they received no PAH-targeted therapy.

However, the larger number of patients in the untreated group recorded overall survival compared with the drug-treated group, but there was no significant difference in the analysis of respiratory-related death only. The patients in the untreated group experienced more adverse events. As mentioned in the Clinical Course of Results, the events included cardiovascular disease, cancer, suicide and others, which were representative of causes of death in COPD patients [42].

The study results suggested that COPD patients with PH might have very poor prognosis, although their PH was eePAP or less severe and that PH conditions might be associated with adverse events which affected their prognosis.

COPD and PAH symptoms are similar, and thus it is difficult to recognize PH combined with COPD unless PH becomes severe. In the current study as well, it was considered difficult to suspect of relatively mild PH based on patient symptoms and clinical assessment of lung disease when looking through blood test, lung function test and changes in HRQoL scores. Authors have already confirmed that COPD patients have RV strain which affects right heart function in the stage of no PH.

In the present study in COPD patients with relatively mild PH, when comparing baseline values with those at month 6 to examine right heart function using TTE, not only pulmonary artery acceleration time (PA AcT) reflecting decline in PAP in the conditions without significant changes in RV performance but also improved RV diastolic performance (decrease of IRT) together with increased RV ejection efficiency (ET elevation) were observed. As a result, the improvement of RV function itself (decrease in Tei index) was noted.

As previously described, we have already confirmed that COPD patients have RV strain which affects right heart function in the stage of no PH [31]. In addition, we have already reported that it is estimated that PAH-targeted therapy for PH helps improve the prognosis in PH patients by alleviating RV strain, leading to improvement of right heart function rather than decrease in PAP [26, 31].

Increase in PAP occurs when right heart itself cannot absorb the pressure in the pulmonary artery. This study also suggests that bosentan, PAH-targeted therapy, improves diastolic filling by decreasing PAP and alleviating RV strain, leading to improvement in RV function itself as well as ejection efficiency.

A small, randomized, controlled study of bosentan in COPD patients with PH reports worsening of gas exchange, lower degree of improvement in maximal oxygen consumption, exercise function and QOL. On the other hand, another study reports that bosentan improves exercise function in COPD-PH. patients. There are still scarce data supporting strongly the effects of ERAs on the pulmonary hemodynamics and exercise tolerance.

It is controversial whether specific PAH therapies such as pharmacotherapy including ERAs are effective in COPD-PH patients. Thus, its efficacy remains unclear [17, 18].

This study has several limitations. Firstly, the sample size of this study was small. The small sample size and the presence of an individual with an extremely low METS in the untreated group at the start of observation led to a lower average METS in the untreated group. While there was no statistically significant difference in METS between the two groups, this reflects the statistical fragility arising from the small sample size. However, we had no sufficient information to determine the sample size, but an earlier bosentan repeated-dose study (AC-052–111 trial) of patients with PAH (WHO functional class III or above) conducted in Japan provided the rationale for the sample size required (i.e., 11 patients required to conduct a two-sided t-test for AUC with two-sided significance level of 5% and 90% power. Given the current state of clinical trials in this field, the sample size of this study appeared never to be too small.

Secondly, the possible imbalance in patient characteristics between the two groups may have affected the study results. However, this study adhered to randomization with the envelope method, thus making such possibility rather unlikely.

Thirdly, in this study, as the clinical course of COPD patients with eePAP or less severe PH untreated with bosentan was very poor, most of them were unable to undergo routine examination. Accordingly, some of them might have had a rapid RAP elevation, while most of the patients undergoing routine examination might have had no rapid RAP elevation, Thus, it is difficult to confirm whether it is true or not.

In actual clinical practice, it is difficult to distinguish symptoms of pulmonary hypertension (PH) from those of lung disease, making it challenging to detect PH complicating lung disease. There is a possibility that exacerbation events of lung disease and exacerbation events of PH are overlooked due to this difficulty in differentiation. Furthermore, as suggested by previous reports [16], analyzing the relationship between hospitalizations and relatively mild PH, not targeting severe PH, was extremely challenging. In fact, in this clinical trial, the causes determining hospitalization and prognosis in both the bosentan treatment group and the non-treatment group were events typical of the clinical course of COPD patients, such as infection, myocardial infarction, and pulmonary thromboembolism (PTE). The results of this trial suggest that early intervention for PH, a prognostic factor for COPD, a systemic disease, may potentially mitigate the adverse effects on COPD patients. However, we cannot make a definitive statement. Considering the results of this study, we believe it serves as a starting point for proposing further detailed clinical research.

Fourthly, as the aim of this study was to observe the clinical course of COPD patients with exercise-induced elevation of PAP or less severe PH and to investigate the impact of bosentan treatment, oxygen was administered as needed at rest or during 6MWT, which is a routine procedure in the real-world clinical course. Thus, this is estimated to be a factor which may have hampered a significant difference in patient symptoms and changes in arterial blood gases.

Unlike the result, this study appears to suggest that bosentan may delay the overtime decrease in 6MWD in which effect of hypoxia is unlikely to develop, because LTOT is introduced to maintain SpO2 ≥ 90% at rest and during 6MWT as needed. Also, the study result appeared to indicate that bosentan significantly suppressed the decrease in maximum exercise tolerance (measured by TMET) over time, which was, however, not as clear as shown in 6MWD. We presumed the reason whether 6MWD might induce hypoxia despite sufficient oxygen supply because the exercise tolerance test forced patients to do the maximum exercise, or patient’s respiratory disturbance might cause hypoxia with increasing the amount of exercise. Moreover, increase in oxygen demand was observed earlier in the untreated group than in the drug-treated group.

In addition, the evaluation of left ventricular diastolic function conducted in this study to exclude patients with factors contributing to Heart Failure With Preserved Ejection Fraction (HFpEF) was a simple assessment using cardiac ultrasound tissue Doppler. This method calculated E (blood flow velocity into the left ventricle) divided by e' (velocity of the mitral annulus) and utilized the E/e′ ratio as well as the Isovolumetric Relaxation Time. However, upon reevaluation using the H2PEF score [43], none of the participants scored 4 points or higher, suggesting that the influence of HFpEF elements on this trial is likely low.

Due to the significant impact of pulmonary hypertension complicating COPD (COPD-PH) on the prognosis of COPD patients, interventions for COPD-PH have the potential to improve outcomes. There have been various reports in the past examining treatments for COPD-PH and investigating the effects of administering pulmonary hypertension drugs on COPD patients [44, 45]. Our current study, which evaluates PH in COPD and examines the long-term effects of PH drug therapy from the early stages of mild PH, is relatively scarce. Moreover, based on our previous report that COPD patients already exhibit right heart load before developing PH [31], and cases with right heart load have a poor prognosis, along with the background of reporting the efficacy of Bosentan treatment for pulmonary hypertension complicating idiopathic pulmonary fibrosis (IPF-PH) from an early stage [26], this specific study population was chosen for the current research.

Again, in this real-world study, the clinical course in the COPD patients with eePAP as nearly-ePH or less severe PH untreated with bosentan was very poor, while it was evidently better in the drug-treated group.

However, for COPD patients with eePAP or less severe PH presenting with respiratory symptoms who had not required for any change of treatment within 3 months prior to study enrollment and whose symptoms were stable but remained and gradually progressed even after COPD therapy, it is still unclear whether bosentan continues to have favourable effect on the clinical course even after the observation period as well if the treatment is continued or whether dose reduction should be made or it should be discontinued.

## Conclusions

This is a rare type of study, because it identified COPD patients with PH which was not severe by conducting proactive testing and bosentan was used and assessed.

The study appears to suggest that the drug-treated group has remarkably better prognosis than that of the untreated group.

The study results suggest that COPD patients with PH may have very poor prognosis, although PH is exercise-induced or mild to moderate. Bosentan appears to improve their prognosis and suppress ADL deterioration without worsening respiratory failure. The small number of subjects in this study, coupled with its reliance on real clinical data, made it inappropriate to report based on the consideration of normal distribution. Further research is required to investigate the association between PH conditions affecting the prognosis and adverse events as well as the impact of PAH-targeted therapy on the clinical course.

### Supplementary Information

**Additional file 1. **Supplementary data on determination of sample size.

**Additional file 2. **Parameters.

**Additional file 3: Supplementary Figure 1. **Schema of color Doppler echocardiographic measurements.

**Additional file 4: Supplementary Figure 2. **TMET(Treadmill exercise test) protocol.

**Additional file 5: Supplementary Figure 3. **Schedule for evaluation of parameters in this study.

**Additional file 6. **Study drug.

**Additional file 7.** Supplementary Clinical course.

**Additional file 8: Supplementary Figure 4.** Assessment of time-course changes in (airflow obstruction indices of pulmonary function parameters) in drug-treated patients with eePAP or less severe PH.

**Additional file 9: Supplementary Figure 5.** Changes in RHC parameters from baseline a: Changes in parameters obtained by RHC from baseline in the drug-treated group for 2 years.

**Additional file 10: Supplementary Table 1.** Maximum exercise tolerance changes over 2 years in untreated group with treated group.

**Additional file 11. **Supplementary results for other parameters.

**Additional file 12. Supplementary Figure 6.** Changes in TTE parameters from baseline to month 6.

**Additional file 13. **Supplementary data on procedures for informed consent.

**Additional file 14. **Supplementary information regarding compensation in case of trial-related injury or death.

**Additional file 15. **Supplementary information regarding medical expenses.

**Additional file 16. **Supplementary information on Guidance for Tracleer Tablets® dosage modification.

**Additional file 17. **Supplementary file Bosentan-treated and non-treated patient.

**Additional file 18. **Supplementary patient enrollment.

## Data Availability

The datasets generated during and/or analyzed during the current study are not publicly available to protect research subject privacy and confidentiality, but are available from the corresponding author on reasonable request.

## Abbreviations

COPD: Chronic obstructive pulmonary disease
ABG: Arterial blood gas
ADL: Activities of daily living
HRQoL: Health related quality of life
BNP: Brain natriuretic peptide
CCO: Continuous cardiac output
CI: Cardiac index
CO: Cardiac output
DPAOE: Diastolic PAP on exercise
ECG: Echocardiography
LTOT: Long-term oxygen therapy
Mpap: Mean pulmonary arterial pressure
mPAPOE: Mean pulmonary arterial pressure on exercise
6MWT: 6-Minute walk test
6 MW: 6-Minute walk distance
PAH: Pulmonary arterial hypertension
PAP: Pulmonary arterial pressure
PaO_2_: Partial pressure of oxygen in arterial blood
PH: Pulmonary hypertension
PAWP: Pulmonary artery wedge pressure
PDE-5: Phosphodiesterase type 5
PVR: Pulmonary vascular resistance
RAP: Right arterial pressure
RVP: Right ventricular pressure
SPAPOE: Systolic pulmonary arterial pressure on exercise
HPV: Hypoxic pulmonary vasoconstriction
