# Supplementary material for: Clinical course of COPD patients with exercise-induced elevation of pulmonary artery pressure or less severe pulmonary hypertension presenting with respiratory symptoms and the impact of bosentan intervention—prospective, single-center, randomized, parallel-group study
Source: BMC Pulm Med. 2024 Feb 17;24:90. doi: 10.1186/s12890-024-02895-0 (PMC10873998; doi:10.1186/s12890-024-02895-0)
Supplement: Supplementary file 1 — Additional file 1. Supplementary data on determination of sample size. [file 12890_2024_2895_MOESM1_ESM.docx]

**Supplementary data on determination of sample size**

With this study still at an exploratory stage, no sufficient information is available to determine the sample size. However, in a repeated-dose study of bosentan (AC-052-111 trial) in patients with PAH (WHO functional class III or above) conducted in Japan, 11 patients were required to perform a two-sided t-test (two-sided significance level of 5%, 90% power) for AUC. In light of this information, we conducted this study in order to evaluate feasibility, time, cost, adverse events, and effect size in an attempt to predict an appropriate sample size and improve upon the study design prior to performance of a full-scale research project based on the number of patients currently available for analysis and we prepared this report an obvious significant difference was already observed in prognosis between the groups compared in this study.
