# Supplementary material for: Clinical course of COPD patients with exercise-induced elevation of pulmonary artery pressure or less severe pulmonary hypertension presenting with respiratory symptoms and the impact of bosentan intervention—prospective, single-center, randomized, parallel-group study
Source: BMC Pulm Med. 2024 Feb 17;24:90. doi: 10.1186/s12890-024-02895-0 (PMC10873998; doi:10.1186/s12890-024-02895-0)
Supplement: Supplementary file 2 — Additional file 2. Parameters. [file 12890_2024_2895_MOESM2_ESM.docx]

**Parameters**

Parameters evaluated included the following:

**Adverse events**

All patients were assessed for adverse events during the run-in period* and every 4 weeks thereafter**, as well as based on their medical records on unscheduled visits to our outpatient clinic. Even for those unable to undergo the periodic assessments due to change of their attending physician, adverse events were assessed by contacting their current physician to have these events confirmed.

**Other parameters**

**Pulmonary function test (PFT)** including spirometry and diffusing capacity of the lungs for carbon monoxide (DLCO) was carried out during the run-in period* and every 6 months thereafter**.

Pulmonary function test (PFT) was carried out during the run-in period* and every 6 months thereafter**. (See also supplementary document on parameters)

**ECG Examination** was carried out during the run-in period* and every 6 months thereafter**. Complete two-dimensional, pulsed-wave, color-flow echocardiography was performed using the Toshiba ultrasound system Xario (TOSHIBA MEDICAL SYSTEMS CORPORATION, Tochigi, Japan) as previously described (26-33).

Right heart circulation was assessed using Color Doppler flow imaging to detect and semi-quantify pulmonary and tricuspid valve regurgitation. Tricuspid inflow velocity was recorded from the apical four-chamber view with the pulsed-wave Doppler sample volume positioned at the tip of the tricuspid leaflets during diastole. The right ventricular outflow tract velocity was recorded from the parasternal short-axis view with the pulsed-wave Doppler sample volume positioned just below the pulmonary valve.

Tricuspid annular plane systolic excursion (TAPSE) was measured as the distance of the right ventricular (RV) tricuspid annular motion from the apical four-chamber view focusing on the RV by pointing the area of interest of M-mode to the valve annulus of the RV lateral wall (34).

By conducting area trace from the apical four-chamber image, the areas and lengths of the right atrium and ventricle were measured during diastole and systole; and further, the RV EF was evaluated based on changes in the RV area.

**Doppler measurements** were carried out during the run-in period* and every 6 months** as follows.

All assessments of RV function were performed according to the methods previously described (26-34) (Supplementary Fig. 1). Mean values were obtained by averaging at least five beats. RV outflow acceleration time (AcT) and tricuspid regurgitation velocity were measured to determine if there was any evidence of PH. PH was considered to be present if the tricuspid regurgitation velocity was > 2.8 m/sec without pulmonary valve stenosis (29,30); if pulmonary artery (PA) AcT was < 90 msec; or if PA AcT/PA ejection time (ET) was < 0.3 (30,31). All measurements were made at end-expiration.

**RHC** was carried out during the run-in period* and every 6 months thereafter**.

Hemodynamic parameters (systolic PAP [SPAP]; diastolic PAP [DPAP]; mean PAP [mPAP]; systolic PAWP [SPAWP]; diastolic PAWP [DPAWP]; mean PAWP [mPAWP]; systolic RVP [SRVP]; diastolic RVP [DRVP]; mean RVP [mRVP]; systolic RAP [SRAP]; diastolic RAP [DRAP]; mean RAP [mRAP]; and cardiac output [CO]) and pulmonary vascular resistance (PVR) were measured with the patient in the supine position via the internal jugular vein and using a Swan-Ganz continuous cardiac output (CCO) thermodilution flow-directed pulmonary artery catheter (Edwards Lifesciences LLC, USA). Cardiac output was measured by the thermodilution method using a Vigilance hemodynamic monitor (Edwards Lifesciences LLC, USA).

Systolic PAP on effort (SPAPOE), diastolic PAP on effort (DPAPOE) and mean PAP on effort (mPAPOE) were measured while patients were clasping and opening both hands repeatedly by putting a full strain on the body. Furthermore, mixed venous blood gas analysis was performed.

**Survival rates:** Hospital-free survival* and overall survival were determined by the duration of survival from week 0 (start of assessment), i.e., as the date treatment started for the drug-treated group and 2 weeks after RHC for the untreated group. Even for those unable to undergo the periodic assessments due to change of their attending physician, etc., this survival analysis was continued by contacting the patient’s current physician to have his/her survival status confirmed. Patients were censored from hospital-free survival if they could no longer continue ambulatory treatment and were admitted to another hospital or if they could no longer present to our hospital for progression of respiratory failure.

**Pulmonary function test (PFT)** including spirometry and diffusing capacity of the lungs for carbon monoxide (DLCO) was carried out during the run-in period* and every 6 months thereafter**.

**Activity and respiratory symptom assessments including exercise tolerance test** (35-38): (See also supplementary document on parameters with Supplementary Figure. 2).

WHO functional class was assessed just before entry and modified Medical Research Council (mMRC) (34), St. George’s Respiratory Questionnaire (SGRQ) (35), Short-Form 36-Item health survey (SF36) (36), 6-minute walk test and exercise tolerance test were performed during the run-in period* and every 6 months thereafter**.

Even for those unable to undergo these periodic assessments due to change of their attending physician or progression of respiratory failure, the mMRC assessment was performed by regularly contacting their current physician unless they developed a new disease unrelated to lung diseases that could affect their ADL.

Activity and respiratory symptom assessments including exercise tolerance test were performed during the run-in period* and every 6 months thereafter**. (See also Supplementary Figure. 2). Those in whom LTOT was in place to ensure adequate oxygen inhalation during 6MWT (deemed equivalent to COPD patients receiving routine therapy in clinical practice to allow them to be monitored for changes in their condition, prognosis and functional capacity for ADL) were assessed for treadmill exercise test (TMET) with LTOT in place.

**Six-minute walk test (6MWT)**

The walking speed was determined for each patient. Walk distance (6MWD), post-walk arterial blood gas (ABG) analysis, brain natriuretic peptide (BNP) and/or N-terminal pro-B-type natriuretic peptide (NT-proBNP) and arterial plasma lactate were measured.

**Exercise tolerance test** was carried out using the treadmill exercise test (TMET). TMET was conducted using MAX 1 (Sensor Medics) according to the protocol proposed by the Research Group on Respiratory Failure (MHLW-designated disease) (Supplementary Fig.2) (38). Maximal exercise tolerance, post-TMET ABG, BNP and/or NT-proBNP and arterial plasma lactate were measured. While these assessments were not feasible in those who could no longer continue ambulatory treatment and had to be admitted for progression of respiratory failure, the following assessments were continued unless they had developed any new disease that could further affect their ADL.

Patients were rated as 4 on the mMRC scale and 0 m in 6MWT if they were found incapable of walking at all; their TMET was rated as 1 METs if they were found incapable of exercising, unless they were confirmed dead; and their TMET was rated as 0 METs if they were found to be in a state of metabolic arrest, unless they had developed any new disease other than respiratory failure or they were confirmed dead.

Those in whom LTOT was in place to ensure adequate oxygen inhalation during 6MWT (deemed equivalent to COPD patients receiving routine therapy in clinical practice to allow them to be monitored for changes in their condition, prognosis and functional capacity for ADL) were assessed for TMET with LTOT in place.

**ABG, arterial plasma lactate,**

Arterial blood gas (ABG), arterial plasma lactate, brain natriuretic peptide (BNP) and/or N-terminal (NT)-proBNP at rest, post-6MWT and post-TMET were determined during the run-in period* and every 6 months thereafter**.

Hematology, biochemistry and urinalysis were performed during the run-in period* and every 4 weeks thereafter**.

*Run-in period: Within 2 weeks after written informed consent was obtained from each patient

**Every 6 months: Consecutive 6 months with a ± 1-week window counting from week 0 (start of assessment) defined as the date drug treatment started for the drug-treated group and 2 weeks after RHC for the untreated group. However, the periodic assessments not conducted in patients as planned based on the attending physician’s judgement were deemed acceptable, unless they met any of the criteria for discontinuation of the study (e.g., pneumonia, etc.) (Fig.1 and Supplementary Fig. 3)

**Hematology, biochemistry and urinalysis** were performed during the run-in period* and every 4 weeks thereafter.

**Adverse events:** These were assessed during the run-in period* and every 4 weeks thereafter, as well as based on patient’s medical records on unscheduled visits to our outpatient clinic. Even for those unable to undergo the periodic assessments due to change of their attending physician, adverse events were assessed by contacting their current physician to have these events confirmed.

***Run-in period:** Within 2 weeks after written informed consent was obtained from each patient

****Every 6 months:** Successive 6 months with a ± 1-week window counting from week 0 (start of assessment) defined as the date drug treatment started for the drug-treated group and 2 weeks after RHC for the untreated group. However, the periodic assessments not conducted in patients as planned based on the attending physician’s judgement were deemed acceptable, unless they met any of the criteria for discontinuation of the study (e.g., pneumonia, etc.) (Fig.1 and Supplementary Fig. 3)
