# Supplementary material for: Clinical course of COPD patients with exercise-induced elevation of pulmonary artery pressure or less severe pulmonary hypertension presenting with respiratory symptoms and the impact of bosentan intervention—prospective, single-center, randomized, parallel-group study
Source: BMC Pulm Med. 2024 Feb 17;24:90. doi: 10.1186/s12890-024-02895-0 (PMC10873998; doi:10.1186/s12890-024-02895-0)
Supplement: Supplementary file 3 — Additional file 3: Supplementary Figure 1. Schema of color Doppler echocardiographic measurements. [file 12890_2024_2895_MOESM3_ESM.pptx]

## Slide 1
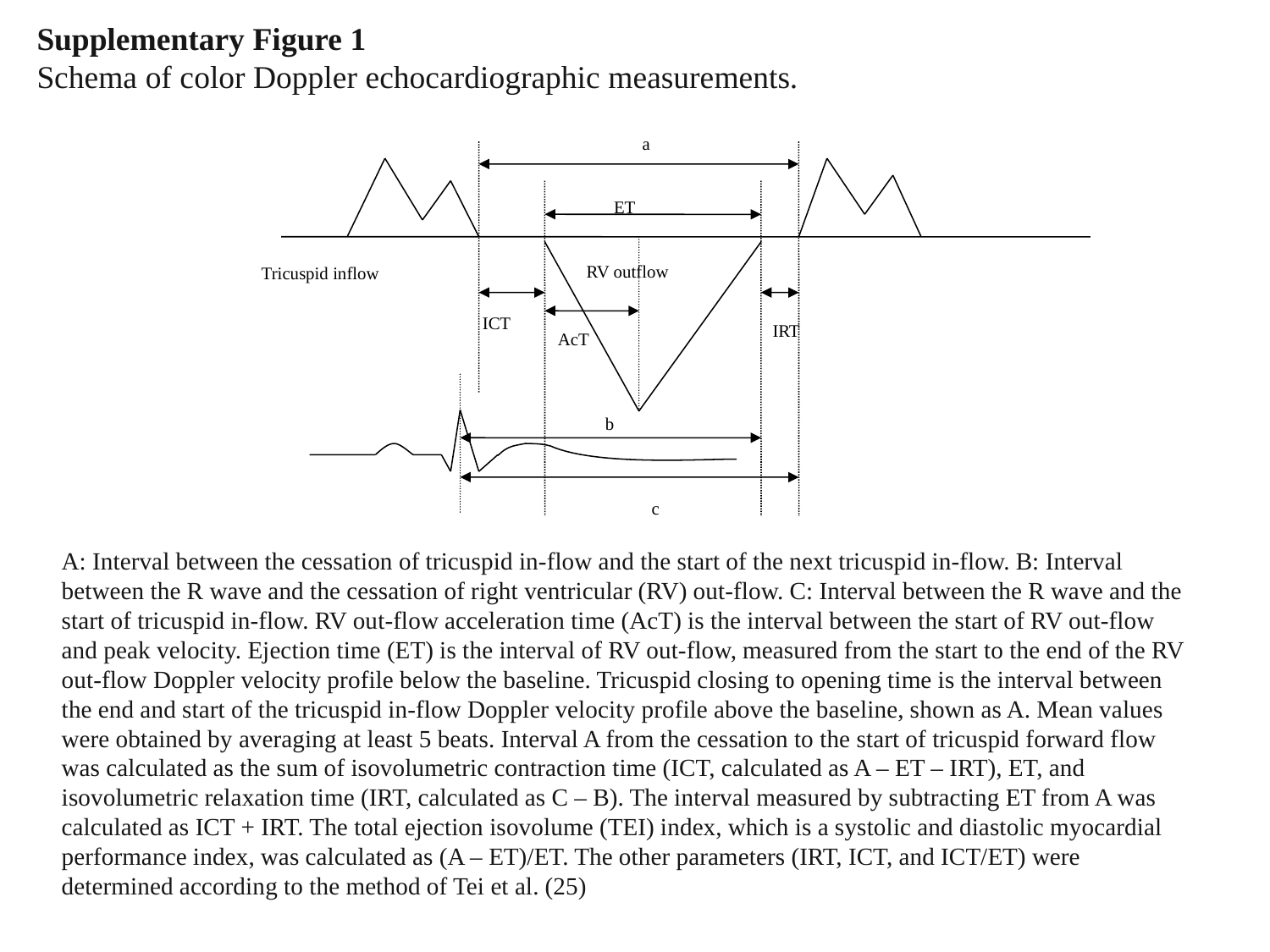

Supplementary Figure 1
Schema of color Doppler echocardiographic measurements.
a
ET
RV outflow
Tricuspid inflow
ICT
IRT
AcT
b
c
A: Interval between the cessation of tricuspid in-flow and the start of the next tricuspid in-flow. B: Interval between the R wave and the cessation of right ventricular (RV) out-flow. C: Interval between the R wave and the start of tricuspid in-flow. RV out-flow acceleration time (AcT) is the interval between the start of RV out-flow and peak velocity. Ejection time (ET) is the interval of RV out-flow, measured from the start to the end of the RV out-flow Doppler velocity profile below the baseline. Tricuspid closing to opening time is the interval between the end and start of the tricuspid in-flow Doppler velocity profile above the baseline, shown as A. Mean values were obtained by averaging at least 5 beats. Interval A from the cessation to the start of tricuspid forward flow was calculated as the sum of isovolumetric contraction time (ICT, calculated as A – ET – IRT), ET, and isovolumetric relaxation time (IRT, calculated as C – B). The interval measured by subtracting ET from A was calculated as ICT + IRT. The total ejection isovolume (TEI) index, which is a systolic and diastolic myocardial performance index, was calculated as (A – ET)/ET. The other parameters (IRT, ICT, and ICT/ET) were determined according to the method of Tei et al. (25)
