# Supplementary material for: Clinical course of COPD patients with exercise-induced elevation of pulmonary artery pressure or less severe pulmonary hypertension presenting with respiratory symptoms and the impact of bosentan intervention—prospective, single-center, randomized, parallel-group study
Source: BMC Pulm Med. 2024 Feb 17;24:90. doi: 10.1186/s12890-024-02895-0 (PMC10873998; doi:10.1186/s12890-024-02895-0)
Supplement: Supplementary file 4 — Additional file 4: Supplementary Figure 2. TMET(Treadmill exercise test) protocol. [file 12890_2024_2895_MOESM4_ESM.pptx]

## Slide 1
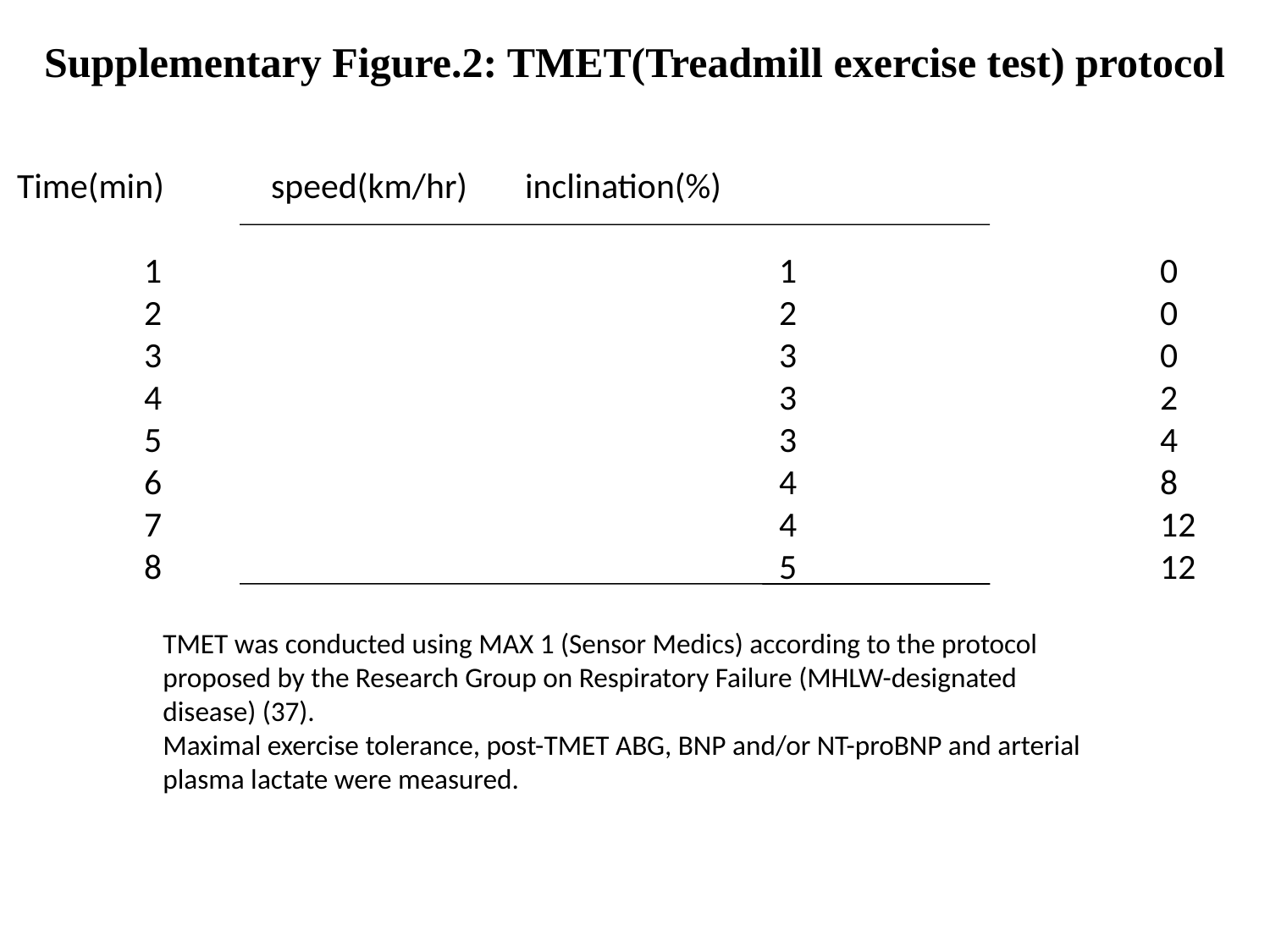

Supplementary Figure.2: TMET(Treadmill exercise test) protocol
Time(min) 	speed(km/hr)	inclination(%)
	1					1		 	0
	2					2		 	0
	3					3		 	0
	4					3		 	2
	5					3			4
	6					4		 	8
	7					4			12
	8					5			12
TMET was conducted using MAX 1 (Sensor Medics) according to the protocol proposed by the Research Group on Respiratory Failure (MHLW-designated disease) (37).
Maximal exercise tolerance, post-TMET ABG, BNP and/or NT-proBNP and arterial plasma lactate were measured.
