# Supplementary material for: Clinical course of COPD patients with exercise-induced elevation of pulmonary artery pressure or less severe pulmonary hypertension presenting with respiratory symptoms and the impact of bosentan intervention—prospective, single-center, randomized, parallel-group study
Source: BMC Pulm Med. 2024 Feb 17;24:90. doi: 10.1186/s12890-024-02895-0 (PMC10873998; doi:10.1186/s12890-024-02895-0)
Supplement: Supplementary file 5 — Additional file 5: Supplementary Figure 3. Schedule for evaluation of parameters in this study. [file 12890_2024_2895_MOESM5_ESM.docx]

**Supplementary Figure 3: Schedule for evaluation of parameters in this study.**

| Item | | Run-in period | Start of treatment | Treatment period (24 months) | | | | | |
| --- | --- | --- | --- | --- | --- | --- | --- | --- | --- |
| Time | | Weeks -0 to -2 | Week 0  (For untreated group, 2 weeks after RHC) | (Week 1) | Week 2 | Week 4 | Week 8 onwards | | |
|  |  |  |  |  |  |  | Every 4 weeks | Every 6 months | At completion (discontinuation) |
| Informed consent | | ○ |  |  |  |  |  |  |  |
| Patient background | | ○ |  |  |  |  |  |  |  |
| Study drug administration  (Drug to treat PAH)* | |  |  |  |  |  |  |  |  |
| Subjective and objective symptoms | | ○ | ○ | ○ | ○ | ○ | ○ | ○ | ○ |
| Monitoring for adverse events | |  |  |  |  |  |  |  |  |
| Blood pressure | | ○ | |  | ○* | ○ | ○ | ○ | ○ |
| Pulse rate | | ○ | |  | ○* | ○ | ○ | ○ | ○ |
| Body weight | | ○ | |  |  |  |  | ○ | ○ |
| Laboratory tests | Hematology | ○ | |  | ○* | ○* | ○ | ○ | ○ |
|  | Biochemistry  (NT-proBNP, etc.) | ○ | |  | ○* | ○* | ○ | ○ | ○ |
|  | Urinalysis | ○ | |  |  | ○* | ○ | ○ | ○ |
| Chest X-ray | | ○ | |  |  | ○ | ○ | ○ | ○ |
| ECG | | ○ | |  |  |  |  | ○ |  |
| Echocardiography | | ○ |  |  |  |  |  | ○ | ○ |
| RHC | | ○ |  |  |  |  |  | ○ |  |
| ADL | | ○ | ○ |  |  |  |  | ○ | ○ |
| Exercise stress testing | | ○ |  |  |  |  |  | ○ | ○ |
| PFT | | ○ | |  |  |  |  | ○ | ○ |
| Arterial blood analysis | | ○ | |  |  |  |  | ○ | ○ |

*To be conducted only in patients given the study drug
