# Supplementary material for: Clinical course of COPD patients with exercise-induced elevation of pulmonary artery pressure or less severe pulmonary hypertension presenting with respiratory symptoms and the impact of bosentan intervention—prospective, single-center, randomized, parallel-group study
Source: BMC Pulm Med. 2024 Feb 17;24:90. doi: 10.1186/s12890-024-02895-0 (PMC10873998; doi:10.1186/s12890-024-02895-0)
Supplement: Supplementary file 6 — Additional file 6. Study drug. [file 12890_2024_2895_MOESM6_ESM.docx]

**Study drug**

Bosentan was administered, as a rule, according to the approved dosage and administration. Bosentan is to be usually initiated in adults at a dose of 62.5 mg twice daily after breakfast and dinner for 4 weeks and increased to a dose of 125 mg twice daily after breakfast and dinner from week 5 of treatment onwards with the dosage adjusted according to the patient’s symptoms and tolerability, but not exceeding 250 mg per day. In this study conducted in routine clinical settings, however, it was acceptable to continue treatment at the initial dosage if deemed by the investigator to be appropriate based on the patient’s condition.

**Supplementary information on Guidance for Tracleer Tablets® dosage modification**

If a patient receiving Tracleer experiences an AST (GOT) or ALT (GPT) elevation greater than 3-fold the upper limit of the normal reference range, dosage modification and liver function tests will be conducted with reference to the guidance provided below:

AST (GOT)/ ALT (GPT): Action taken with Tracleer therapy and frequency of liver function tests

>3-fold and ≤ 5-fold ULN The dosage of Tracleer should be reduced or the therapy with the drug discontinued. Thereafter, AST and ALT should be measured at least every 2 weeks. If the values return to baseline, Tracleer therapy may be continued or resumed as appropriate.*

>5-fold and ≤8-fold ULN Tracleer therapy should be discontinued. Thereafter, AST and ALT should be measured at least every 2 weeks. If the values return to baseline, resumption of Tracleer therapy may be considered.*

>8-fold ULN Tracleer therapy should be discontinued and should not be resumed.

ULN: Upper limit of normal

* To resume Tracleer therapy, the starting dose should be used. After the therapy is resumed, AST and ALT values should be measured again within 3 days, and then at 2 weeks from the date the therapy is resumed. Subsequently, the therapy is given again in accordance with the above guidance for dosage adjustment and liver function tests.
