# Supplementary material for: Clinical course of COPD patients with exercise-induced elevation of pulmonary artery pressure or less severe pulmonary hypertension presenting with respiratory symptoms and the impact of bosentan intervention—prospective, single-center, randomized, parallel-group study
Source: BMC Pulm Med. 2024 Feb 17;24:90. doi: 10.1186/s12890-024-02895-0 (PMC10873998; doi:10.1186/s12890-024-02895-0)
Supplement: Supplementary file 7 — Additional file 7. Supplementary Clinical course. [file 12890_2024_2895_MOESM7_ESM.docx]

See Supplementary Clinical course

Eight of the 15 untreated patients received LTOT. Of the 15 patients, 3 completed the 2-year treatment period, 6 were not available for the periodic assessments from day 38, day 64, day 125, day 270, day 606 and day 286 due to any reason (PTE; aortic aneurysm; cerebral infarction; acute myocardial infarction; no RHC at month 18 and unable to stay at home as well as hospital transfer and withdrawal from the study assessment because of the patient wish; and treated as discontinuation due to suicide, respectively). Of the remaining 6 patients, 2 were censored from hospital-free survival analysis at day 49 and day 490 due to progression of respiratory failure and censored from overall survival at day 94 and day 541 due to progression of respiratory failure, 1 was censored from hospital-free survival analysis at day 369 due to respiratory failure associated with pneumothorax. Regarding the data after starting to record hospital-free survival, the patient was confirmed alive until day 730 and received the same treatment after transferring to another hospital. 1 was censored from hospital-free survival analysis at day 151 and overall survival at the same time due to acute myocardial infarction, 1 was censored from hospital-free survival at day 368 and overall survival analysis at the same time due to bleeding caused by cervical cancer, and 1 was censored from hospital-free survival analysis at day 610 and censored from overall survival at day 623 due to sepsis secondary to urinary tract infection. No patient discontinued the treatment for other reasons than any adverse event. The data on patients whom observation was discontinued were recorded until the time of discontinuation. For the other patients, data of 6MWT and TMET were recorded for 2 years. In the analysis, 6MWT was recorded as 0 m after a patient became bedridden or died, and TMET was recorded as 1MET after a patient became bedridden and 0MET after a patient died excluding suicide patient. Other parameters were recorded until hospital-free survival or all data were recorded during the 2-year observation period.

Eight of the 14 bosentan-treated patients included in the analysis received LTOT. Of all, nine patients completed the study after finishing the assessments at month 24. Of the remaining 5 patients, one patient was transferred to another hospital at day 591 but continued the treatment and assessed at month 24 at our hospital. All data excluding adverse events after day 591 were recorded. One withdrew from the study because of difficult hospital visit at day 473 due to disc hernia, 1 withdrew from the study because of transferring to another hospital due to moving, 1 was censored from hospital-free survival analysis at day 727 and overall survival analysis at the same time due to sudden death possibly due to aplastic anemia, and 1 was censored from hospital-free survival analysis at day 153 due to leg strength declines associated with breathlessness, and after starting to record hospital-free survival, 6MWT was recorded as 0 m after transferring to another hospital and continuing the same treatment and TMET was recorded as 1MET and confirmed alive until day 730.

Based on these findings, the clinical course in the untreated group was much poorer than in the treated group. Only quite a few patients could be assessed after month 12 in the current study excluding those patients with 1MET indicating bedridden and 6MWT=0 m during the period since recording hospital-free survival (i.e., bedridden) and with 0MET indicating death and 6MWT=0 m during the period since recording overall survival.
