# Supplementary material for: Clinical course of COPD patients with exercise-induced elevation of pulmonary artery pressure or less severe pulmonary hypertension presenting with respiratory symptoms and the impact of bosentan intervention—prospective, single-center, randomized, parallel-group study
Source: BMC Pulm Med. 2024 Feb 17;24:90. doi: 10.1186/s12890-024-02895-0 (PMC10873998; doi:10.1186/s12890-024-02895-0)
Supplement: Supplementary file 8 — Additional file 8: Supplementary Figure 4. Assessment of time-course changes in (airflow obstruction indices of pulmonary function parameters) in drug-treated patients with eePAP or less severe PH. [file 12890_2024_2895_MOESM8_ESM.pptx]

## Slide 1
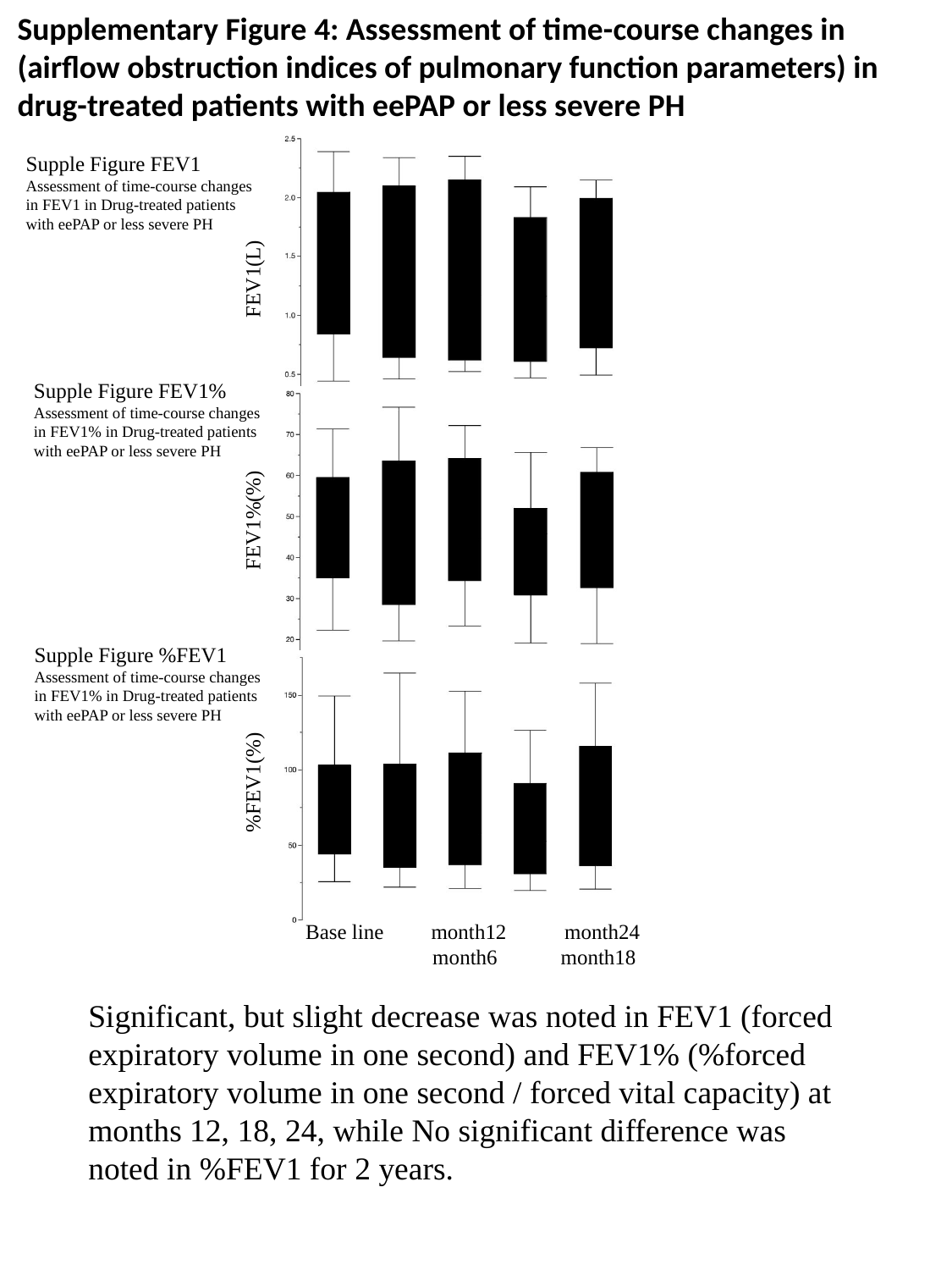

Supplementary Figure 4: Assessment of time-course changes in (airflow obstruction indices of pulmonary function parameters) in drug-treated patients with eePAP or less severe PH
Supple Figure FEV1
Assessment of time-course changes
in FEV1 in Drug-treated patients
with eePAP or less severe PH
FEV1(L)
Supple Figure FEV1%
Assessment of time-course changes
in FEV1% in Drug-treated patients
with eePAP or less severe PH
FEV1%(%)
Supple Figure %FEV1
Assessment of time-course changes
in FEV1% in Drug-treated patients
with eePAP or less severe PH
%FEV1(%)
Base line month12 month24
	month6 month18
Significant, but slight decrease was noted in FEV1 (forced expiratory volume in one second) and FEV1% (%forced expiratory volume in one second / forced vital capacity) at months 12, 18, 24, while No significant difference was noted in %FEV1 for 2 years.
