# Supplementary material for: Clinical course of COPD patients with exercise-induced elevation of pulmonary artery pressure or less severe pulmonary hypertension presenting with respiratory symptoms and the impact of bosentan intervention—prospective, single-center, randomized, parallel-group study
Source: BMC Pulm Med. 2024 Feb 17;24:90. doi: 10.1186/s12890-024-02895-0 (PMC10873998; doi:10.1186/s12890-024-02895-0)
Supplement: Supplementary file 9 — Additional file 9: Supplementary Figure 5. Changes in RHC parameters from baseline a: Changes in parameters obtained by RHC from baseline in the drug-treated group for 2 years. [file 12890_2024_2895_MOESM9_ESM.pptx]

## Slide 1
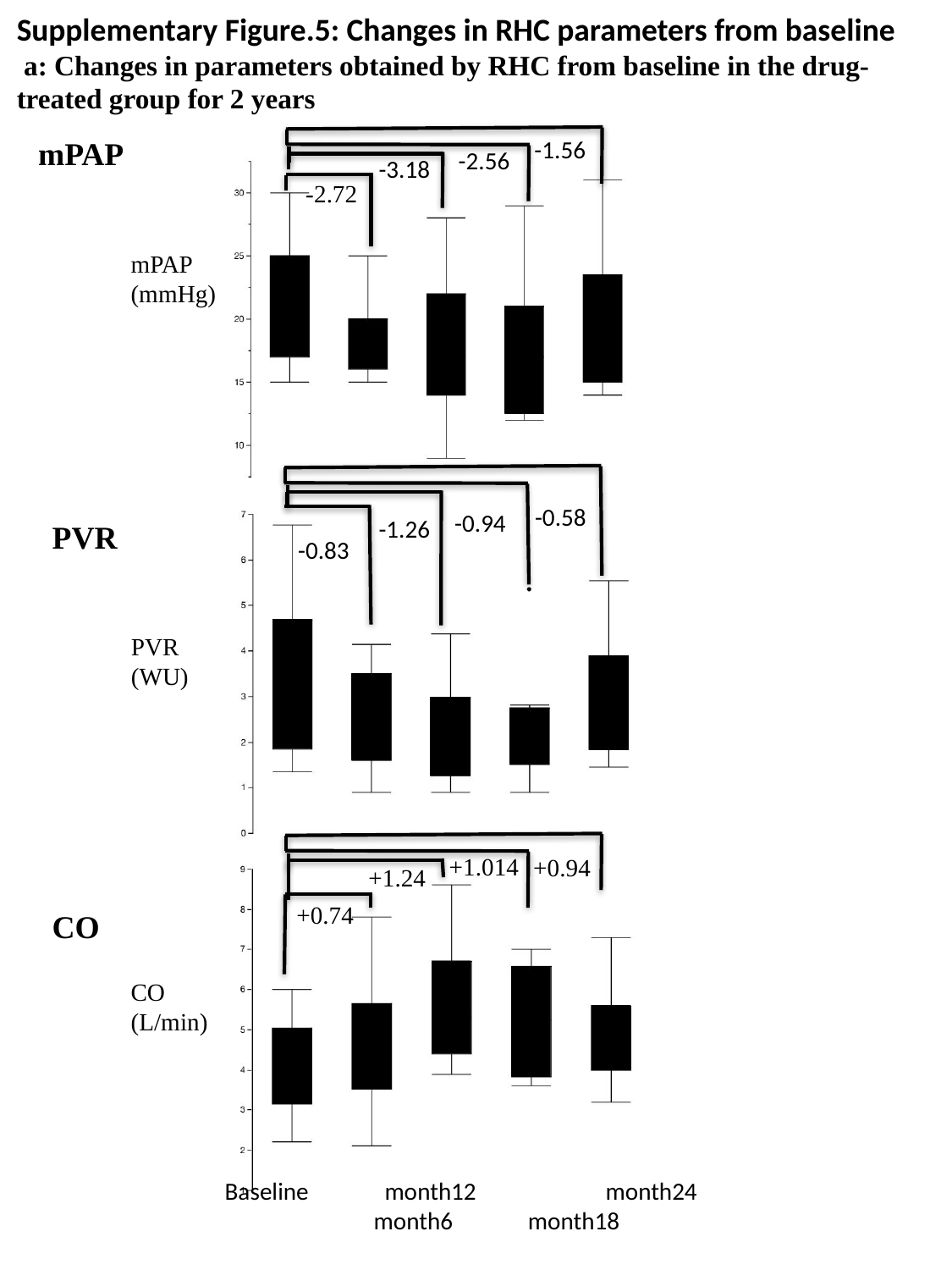

Supplementary Figure.5: Changes in RHC parameters from baseline
 a: Changes in parameters obtained by RHC from baseline in the drug-treated group for 2 years
mPAP
-1.56
-2.56
-3.18
-2.72
mPAP
(mmHg)
-0.58
-0.94
-1.26
PVR
-0.83
PVR
(WU)
+1.014
+0.94
+1.24
+0.74
CO
CO
(L/min)
Baseline 	 month12	 	month24
	 month6 	 month18

## Slide 2
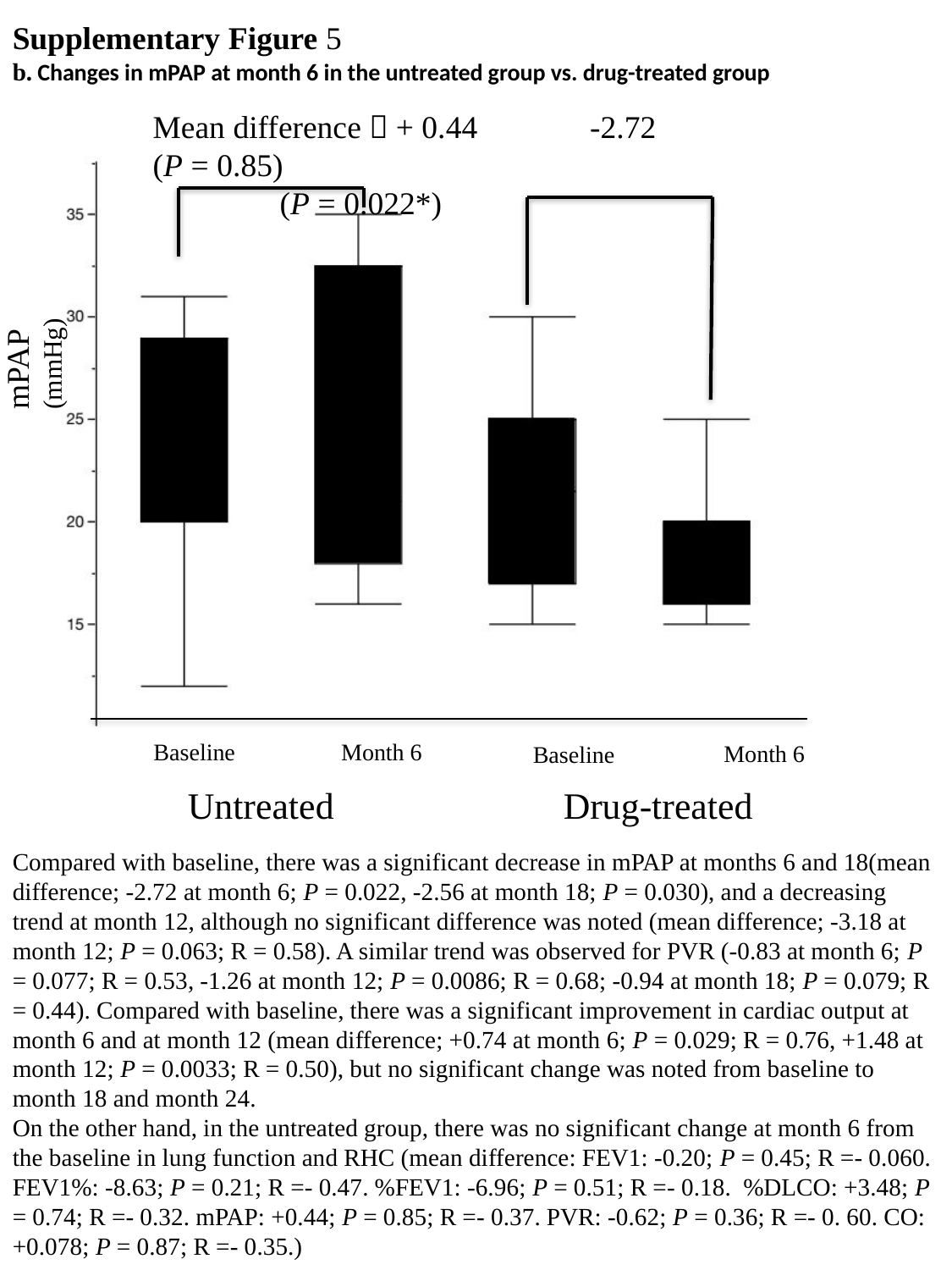

Supplementary Figure 5
b. Changes in mPAP at month 6 in the untreated group vs. drug-treated group
Mean difference：+ 0.44 -2.72
(P = 0.85)					(P = 0.022*)
mPAP
(mmHg)
Month 6
Baseline
Month 6
Baseline
Untreated
Drug-treated
Compared with baseline, there was a significant decrease in mPAP at months 6 and 18(mean difference; -2.72 at month 6; P = 0.022, -2.56 at month 18; P = 0.030), and a decreasing trend at month 12, although no significant difference was noted (mean difference; -3.18 at month 12; P = 0.063; R = 0.58). A similar trend was observed for PVR (-0.83 at month 6; P = 0.077; R = 0.53, -1.26 at month 12; P = 0.0086; R = 0.68; -0.94 at month 18; P = 0.079; R = 0.44). Compared with baseline, there was a significant improvement in cardiac output at month 6 and at month 12 (mean difference; +0.74 at month 6; P = 0.029; R = 0.76, +1.48 at month 12; P = 0.0033; R = 0.50), but no significant change was noted from baseline to month 18 and month 24.
On the other hand, in the untreated group, there was no significant change at month 6 from the baseline in lung function and RHC (mean difference: FEV1: -0.20; P = 0.45; R =- 0.060. FEV1%: -8.63; P = 0.21; R =- 0.47. %FEV1: -6.96; P = 0.51; R =- 0.18. %DLCO: +3.48; P = 0.74; R =- 0.32. mPAP: +0.44; P = 0.85; R =- 0.37. PVR: -0.62; P = 0.36; R =- 0. 60. CO: +0.078; P = 0.87; R =- 0.35.)
