# Supplementary material for: Clinical course of COPD patients with exercise-induced elevation of pulmonary artery pressure or less severe pulmonary hypertension presenting with respiratory symptoms and the impact of bosentan intervention—prospective, single-center, randomized, parallel-group study
Source: BMC Pulm Med. 2024 Feb 17;24:90. doi: 10.1186/s12890-024-02895-0 (PMC10873998; doi:10.1186/s12890-024-02895-0)
Supplement: Supplementary file 10 — Additional file 10: Supplementary Table 1. Maximum exercise tolerance changes over 2 years in untreated group with treated group. [file 12890_2024_2895_MOESM10_ESM.docx]

Supplementary table**.1.** Maximum exercise tolerance changes over 2 years in untreated group with treated group

Untreated group

|  | baseline | Month6 | Month12 | Month18 | Month24 |
| --- | --- | --- | --- | --- | --- |
| Maximum exercise tolerance (METs)  mean±S.D | 2.39±0.69 | 1.85±1.07 | 2.07±1.77 | 1.23±1.28 | 1.17±1.86 |

Treated group

|  | baseline | Month6 | Month12 | Month18 | Month24 |
| --- | --- | --- | --- | --- | --- |
| Maximum exercise tolerance (METs)  mean±S.D | 4.74±2.78 | 4.56±2.86 | 4.73±2.90 | 4.45±3.11 | 3.72±2.85 |
