# Supplementary material for: Clinical course of COPD patients with exercise-induced elevation of pulmonary artery pressure or less severe pulmonary hypertension presenting with respiratory symptoms and the impact of bosentan intervention—prospective, single-center, randomized, parallel-group study
Source: BMC Pulm Med. 2024 Feb 17;24:90. doi: 10.1186/s12890-024-02895-0 (PMC10873998; doi:10.1186/s12890-024-02895-0)
Supplement: Supplementary file 11 — Additional file 11. Supplementary results for other parameters. [file 12890_2024_2895_MOESM11_ESM.docx]

**Supplementary results for other parameters**

**HRQOL (mMRC, SGRQ, SF-36)**

***Untreated patients with eePAP or less severe PH***

Compared with baseline (Table 1), no significant changes were observed in any HRQOL parameters; given that many of these patients had reached the endpoint of hospital-free survival and thus became unavailable for HRQOL, it was difficult to draw any conclusion on changes over time for 2 years.

***Drug-treated patients with eePAP or less severe PH***

Compared with baseline (Table 1), there was no significant change in mMRC dyspnea throughout the two years.

Since the clinical course was evidently better in the drug-treated-group compared with the untreated group. Also, HRQoL was better in the drug-treated group when compared with baseline score (Table 1). HRQoL data were available in most of the patients for assessment throughout the two years.

However, there was no clinically significant change from baseline.

The details are as follows:

SGRQ symptom (-5.26 at month 6; *P* = 0.49, +2.3 at month 12; *P* = 0.72, +11.79 at month 18; *P* = 0.052, and -2.26 at month 24; *P* = 0.72), SGRQ activity (+5.9 at month 6; *P* = 0.23, +7.75 at month 12; *P* = 0.17, +0.29 at month 18; *P* = 0.96, and +12.04 at 4month 24; *P* = 0.071), SGRQ Impact (+0.92 at month 6; *P* = 0.84, +2.55 at month 12; *P* = 0.60, +3.16 at month 18; *P* = 0.56, and +2.46 at month 24; *P* = 0.68), Total SGRQ (+1.26 at month 6; *P* = 0.77, +1.21 at month 12; *P* = 0.79, +7.089 at month 18; *P* = 0.16, and +4.58 at month 24; *P* = 0.40), SF36PF (-10.42 at month 6; *P* = 0.032, -7.73 at month 12; *P* < 0.050, +0.0 at month 18; *P* = 1.00, and -12.78 at month 2; *P* = 0.075), SF36RP (-15.11 at month 6; *P* = 0.14, -11.93 at month 12; *P* = 0.011, -5.54 at month 18; *P* = 0.49, and -24.30 at month 24; *P* = 0.013), SF36BP (+4.58 at month 6; *P* = 0.58; +1.091 at month 12; *P* = 0.92, +6.44 at month 18; *P* = 0.61, and +3.67 at month 24; *P* = 0.79), SF36GH (+0.83 at month 6; *P* = 0.89, -2.73 at month 12; *P* = 0.62, -2.56 at month 18; *P* = 0.61, and -0.67 at month 24; *P* = 0.89), SF36VT (+2.97 at month 6; *P* = 0.70, -1.69 at month 12; *P* = 0.77, +5.90 at month 18; *P* = 0.16, and -8.32 at month 24; *P* = 0.24); SF36SF (+8.14, at month 6; *P* = 0.50; +1.14 at month 12; *P* = 0.83, 0.00 at month 18; *P* = 1.00, and -4.17 at month 24; *P* = 0.64), SF36RE (-10.41 at month 6; *P* = 0.11; -17.43 at month 12; *P* = 0.11, -17.71 at month 18; *P* = 0.26, and -25.92 at month 24; *P* = 0.12), SF36MH (+4.58 at month 6,; *P* = 0.48; +1.027 at month 12; *P* = 0.92, +4.44 at month 18; *P* = 0.74, and -2.22 at month 24; *P* = 0.85).

Moreover, there was a significant difference between the 2 groups in the change in mMRC from baseline to month 6 (*P* = 0.034) (Supple Figure ADLa).

**TTE**

Although in the untreated group, it was difficult to draw any conclusion because of few data available for analysis due to the small number of patients, no significant change was observed in any of the parameters assessed. In the drug-treated patients, there was a significant change observed especially at month 6 from baseline (Table 1) (mean difference: ET: +23.85; *P* = 0.0048. PA AcT: +21.46; *P*=0.0023. IRT: -27.31; *P*=0.0095. ICT: +2.46; *P*=0.70. TEI index: -0.11; *P*=0.041) (supple Fig.6)

**Other parameters**

***Untreated patients with eePAP or less severe PH***

Although it was difficult to draw any conclusion from the small number of patients currently available for analysis, no significant change was observed in any of the parameters assessed.

***Drug-treated patients with eePAP or less severe PH***

**Laboratory data (blood sampling at rest)**

Compared with baseline (Table 1b), there was no clinically significant change in AGB parameters. Compared with baseline, PO_2_ at month 18 showed a decrease with a mean difference of -13.69 (*P* = 0.030), there was no significant difference at month 24. Thus, no clinical problem was raised.

**Post-6MWT and Post-TMET**

Compared with baseline, no significant change in AGB data was noted throughout the two years.
