# Supplementary material for: Clinical course of COPD patients with exercise-induced elevation of pulmonary artery pressure or less severe pulmonary hypertension presenting with respiratory symptoms and the impact of bosentan intervention—prospective, single-center, randomized, parallel-group study
Source: BMC Pulm Med. 2024 Feb 17;24:90. doi: 10.1186/s12890-024-02895-0 (PMC10873998; doi:10.1186/s12890-024-02895-0)
Supplement: Supplementary file 12 — Additional file 12. Supplementary Figure 6. Changes in TTE parameters from baseline to month 6. [file 12890_2024_2895_MOESM12_ESM.pptx]

## Slide 1
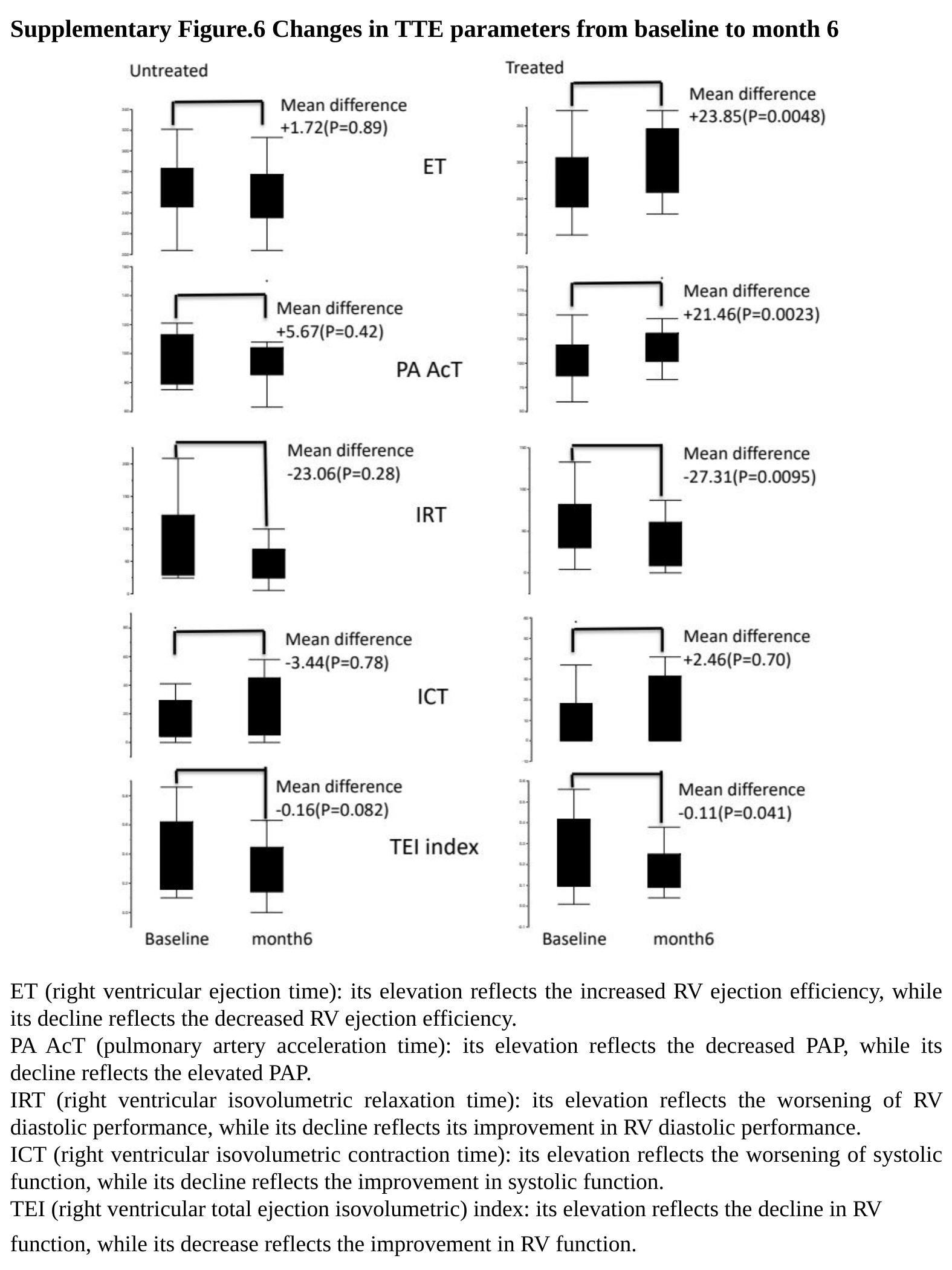

Supplementary Figure.6 Changes in TTE parameters from baseline to month 6
ET (right ventricular ejection time): its elevation reflects the increased RV ejection efficiency, while its decline reflects the decreased RV ejection efficiency.
PA AcT (pulmonary artery acceleration time): its elevation reflects the decreased PAP, while its decline reflects the elevated PAP.
IRT (right ventricular isovolumetric relaxation time): its elevation reflects the worsening of RV diastolic performance, while its decline reflects its improvement in RV diastolic performance.
ICT (right ventricular isovolumetric contraction time): its elevation reflects the worsening of systolic function, while its decline reflects the improvement in systolic function.
TEI (right ventricular total ejection isovolumetric) index: its elevation reflects the decline in RV function, while its decrease reflects the improvement in RV function.
