# Supplementary material for: Clinical course of COPD patients with exercise-induced elevation of pulmonary artery pressure or less severe pulmonary hypertension presenting with respiratory symptoms and the impact of bosentan intervention—prospective, single-center, randomized, parallel-group study
Source: BMC Pulm Med. 2024 Feb 17;24:90. doi: 10.1186/s12890-024-02895-0 (PMC10873998; doi:10.1186/s12890-024-02895-0)
Supplement: Supplementary file 13 — Additional file 13. Supplementary data on procedures for informed consent. [file 12890_2024_2895_MOESM13_ESM.docx]

**Supplementary data on procedures for informed consent**

An informed consent form describing the following items was prepared. Consent had to be obtained in writing.

1. Introduction: About target disease and Tracleer Tablets®

2. Purpose of the study

3. Study methods

4. Planned duration of your participation in this study

5. Planned number of patients participating in this study

6. Anticipated benefits and possible side effects of the study drug

7. Alternative treatment methods

8. Health injury during this study

9. Voluntary participation in this study

10. Prompt notification of information related to the study drug

11. Possible discontinuation of treatment with the study drug

12. Review of medical records during or after the study

13. Publication and protection of personal data

14. Responsibilities associated with consent to participate in this study

15. Anticipated expenses to be borne by patients

16. Study center

17. Attending physician and medical consultation
