# Supplementary material for: Clinical course of COPD patients with exercise-induced elevation of pulmonary artery pressure or less severe pulmonary hypertension presenting with respiratory symptoms and the impact of bosentan intervention—prospective, single-center, randomized, parallel-group study
Source: BMC Pulm Med. 2024 Feb 17;24:90. doi: 10.1186/s12890-024-02895-0 (PMC10873998; doi:10.1186/s12890-024-02895-0)
Supplement: Supplementary file 17 — Additional file 17. Supplementary file Bosentan-treated and non-treated patient. [file 12890_2024_2895_MOESM17_ESM.docx]

Supplementary file ***Bosentan-treated and non-treated patient.***

***Bosentan-treated and non-treated patients***

All patients who met the eligibility criteria and gave informed consent prior to participating in this study were evaluated for PAP and right heart function. These patients with assuming PAWP ≤ 15mmHg, mPAP ≥ 25 mmHg (at rest) to < 35 mmHg and/or mPAPOE ≥30 mmHg were stratified by mPAP. Patients were randomly allocated to either bosentan (drug-treated group) or no treatment (untreated group) by the envelope method.

Both the drug-treated and untreated group comprised those who were diagnosed at this hospital as having COPD without hypoxia (PaO_2_ > 60 mmHg) during the 6-minute walk and who gave informed consent to participate in this study after PAP and right heart function assessments. This group included those with eePAP or less severe PH (mPAP ≥ 25 mmHg [at rest] to < 35 mmHg and/or mPAPOE ≥30 mmHg). (**Fig.1**).

The study required that COPD patients be randomized to drug-treated and untreated groups to investigate their clinical course in real-world settings, with no change of treatment allowed including bosentan for 2 years or until their death as a rule, except for minimal symptomatic therapy (including oxygen volume adjustments required to ensure similar oxygen conditions among the patients), which met none of the exclusion criteria.
