# Supplementary material for: Clinical course of COPD patients with exercise-induced elevation of pulmonary artery pressure or less severe pulmonary hypertension presenting with respiratory symptoms and the impact of bosentan intervention—prospective, single-center, randomized, parallel-group study
Source: BMC Pulm Med. 2024 Feb 17;24:90. doi: 10.1186/s12890-024-02895-0 (PMC10873998; doi:10.1186/s12890-024-02895-0)
Supplement: Supplementary file 18 — Additional file 18. Supplementary patient enrollment. [file 12890_2024_2895_MOESM18_ESM.docx]

Supplementary patient enrollment

54 COPD patients with respiratory symptoms as a chief complaint requiring no change of treatment within 3 months prior to study enrollment and whose symptoms were stable but remained and gradually progressed even after COPD therapy (e.g., long-acting muscarinic antagonist [LAMA] and/or long-acting beta-agonist (LABA) and inhaled steroid [ICS] as needed) and who did not meet any of the exclusion criteria after undergoing all available tests at our hospital were assessed for study eligibility. Of all, a total of 29 patients who underwent RHT for detail examination were enrolled in the current study between August 2010 and October 2018. They were all outpatients who had met the inclusion criteria. At the time of their initial presentation to our hospital, all patients were confirmed to have COPD based on pulmonary function test after inhaled bronchodilator. (Figure.1).

Of these 29 patients, 9 (including 1 female) had eePAP with mPAP < 20mmHg at rest and 20 patients had less severe PH. All of them were randomized to receive or not to receive bosentan therapy. Of these, 14 were in the drug-treated group and the other 15 were in the untreated group; 6 in the drug-treated group and 3 in the untreated group confirmed to have eePAP diagnosed as assuming ≤15mmHg, mPAPOE ≥ 30 mmHg and mPAP at rest <20 mmHg; and 8 in the treated group and 12 in the untreated group were confirmed to have nearly eePAP based on mPAP ≤ 20 at rest to < 35mmHg (less severe PH).
